# Microbial Biomarker Transition in High-Altitude Sinter Mounds From *El Tatio* (Chile) Through Different Stages of Hydrothermal Activity

**DOI:** 10.3389/fmicb.2018.03350

**Published:** 2019-01-15

**Authors:** Laura Sanchez-Garcia, Miguel Angel Fernandez-Martinez, Miriam García-Villadangos, Yolanda Blanco, Sherry L. Cady, Nancy Hinman, Mark E. Bowden, Stephen B. Pointing, Kevin C. Lee, Kimberly Warren-Rhodes, Donnabella Lacap-Bugler, Nathalie A. Cabrol, Victor Parro, Daniel Carrizo

**Affiliations:** ^1^Centro de Astrobiología (CSIC-INTA), Madrid, Spain; ^2^Environmental Molecular Sciences Laboratory, Pacific Northwest National Laboratory, Richland, WA, United States; ^3^Department of Geosciences, University of Montana, Missoula, MT, United States; ^4^Yale-NUS College, National University of Singapore, Singapore, Singapore; ^5^School of Science, Auckland University of Technology, Auckland, New Zealand; ^6^SETI Institute, Mountain View, CA, United States; ^7^NASA Ames Research Center, Moffett Field, CA, United States

**Keywords:** lipid biomarkers, microbial transition, hydrothermal activity, sinter mounds, high altitude geyser field, biogeochemical reconstruction

## Abstract

Geothermal springs support microbial communities at elevated temperatures in an ecosystem with high preservation potential that makes them interesting analogs for early evolution of the biogeosphere. The *El Tatio* geysers field in the Atacama Desert has astrobiological relevance due to the unique occurrence of geothermal features with steep hydrothermal gradients in an otherwise high altitude, hyper-arid environment. We present here results of our multidisciplinary field and molecular study of biogeochemical evidence for habitability and preservation in silica sinter at *El Tatio*. We sampled three morphologically similar geyser mounds characterized by differences in water activity (*i.e.*, episodic liquid water, steam, and inactive geyser lacking hydrothermal activity). Multiple approaches were employed to determine (past and present) biological signatures and dominant metabolism. Lipid biomarkers indicated relative abundance of thermophiles (dicarboxylic acids) and sulfate reducing bacteria (branched carboxylic acids) in the sinter collected from the liquid water mound; photosynthetic microorganisms such as cyanobacteria (alkanes and isoprenoids) in the steam sinter mound; and archaea (squalane and crocetane) as well as purple sulfur bacteria (cyclopropyl acids) in the dry sinter from the inactive geyser. The three sinter structures preserved biosignatures representative of primary (thermophilic) and secondary (including endoliths and environmental contaminants) microbial communities. Sequencing of environmental 16S rRNA genes and immuno-assays generally corroborated the lipid-based microbial identification. The multiplex immunoassays and the compound-specific isotopic analysis of carboxylic acids, alkanols, and alkanes indicated that the principal microbial pathway for carbon fixation in the three sinter mounds was through the Calvin cycle, with a relative larger contribution of the reductive acetyl-CoA pathway in the dry system. Other inferred metabolic traits varied from the liquid mound (iron and sulfur chemistry), to the steam mound (nitrogen cycle), to the dry mound (perchlorate reduction). The combined results revealed different stages of colonization that reflect differences in the lifetime of the mounds, where primary communities dominated the biosignatures preserved in sinters from the still active geysers (liquid and steam mounds), in contrast to the surviving metabolisms and microbial communities at the end of lifetime of the inactive geothermal mound.

## Introduction

Geothermal springs are natural environments of scientific interest because of their significance in the early evolution of the biogeosphere (Walter and Des Marais, 1993; Konhauser et al., 2003; Cady et al., 2018). Despite their apparent in hospitability, terrestrial geothermal springs are recognized habitats for microbial life on Earth (Brock, 1978). Indeed, they are considered some of the candidate sites where life began (Van Kranendonk et al., 2017), in contrast to the classical sub-marine hydrothermal-vents theory including the alkaline hydrothermal vent model (Russell, 2018). Though surficial hydrothermal vents are characterized by steep geothermal gradients and a perpetual supply of nutrients, geothermal springs also provide an environment in which intermittent wetting and drying of hydrothermal precipitates occurs due to the stochastic nature of surface geothermal activity (Damer and Deamer, 2015). Alternating wet and dry periods of hydrothermal activity promotes the interaction of simple molecular building blocks to form complex molecules (Deamer and Georgiou, 2015). Although not consensus exists on whether wet-dry cycling played a role during abiogenesis (Russell, 2018), the possibility of a land-based origin of life strengthens the relevance of such settings for astrobiological exploration (Van Kranendonk et al., 2017).

Geothermal springs and geysers are manifestations of volcanic or impact activity on a wet rocky planet (Sillitoe, 2015). The interaction of groundwater with solidified but still-hot country rock at shallow depths provides a variety of potential niches for heat-loving microbes. In such geothermal systems, groundwater percolates through fractures in igneous rock deep underground, where heat from the nearby magma chamber heats the pressurized fluid to a temperature above its boiling point at surface pressure. Rising superheated fluid emerges at surface effluents associated with hot spring pools and geysers as a column of hot water and steam that erupts episodically as steam and fluid separate as they rise to surface. Fumaroles are characterized exclusively by steam-driven hydrological activity. Regardless of the surface expression of subsurface hydrothermal activity, the environmental conditions of such settings are extreme. Water and steam temperatures are typically several tens of degrees (that extend to the local temperature of boiling) above mean air temperatures, and the pH of the fluid can range from acidic to alkaline. When the elemental content of hydrothermal fluids is high (*i.e.*, high concentration of dissolved silica, calcium, or carbonates), sinter precipitation occurs. The metals contents (Fe, Mn, and Mg), including toxic metals (*e.g.*, Sb, B, or As) of hydrothermal systems can be abundant. In these extreme environments, only the microorganisms particularly resistant and adapted to adverse conditions are able to thrive. Their distribution and community structures appears to be mostly determined by factors such as temperature, pH, or the content of hydrogen sulfide (*e.g.*, Purcell et al., 2007), as demonstrated by diverse studies on geothermal springs from Yellowstone National Park (Cady and Farmer, 1996), Thailand (Purcell et al., 2007), California and Nevada (Zhang et al., 2007), or New Zealand (*e.g.*, Pantcost et al., 2005; Kaur et al., 2015). Temperature is limiting for certain groups such as phototrophic bacteria (*e.g.*, cyanobacteria, green sulfur and non-sulfur bacteria), as their growth is seriously hampered at temperatures greater than 73°C (Ward et al., 1998; Miller and Castenholz, 2000). Conversely, chemolithoautotrophs such as Aquificales are well adapted to thrive in such high temperatures (Reysenbach et al., 1994; Kato et al., 2004). Moreover, hydrogen sulfide is a well-known inhibiting factor of cyanobacteria (Castenholz, 1973; Giovannoni et al., 1987), while it enhances or necessitates the growth of phototrophic sulfur bacteria (Madigan, 1986; van der Meer et al., 2000) or certain species of Aquificales (Skirnisdottir et al., 2000; Nakagawa and Fukui, 2003; Kato et al., 2004). Taken together, geothermal springs are ideal settings for investigating habitability and adaptability of extremophiles in relation to various environmental conditions (Lacap et al., 2007; Purcell et al., 2007; Lau et al., 2008).

Geothermal springs continuously release mineral-rich fluids that precipitate sinters (typically opaline silica, bicarbonate, or iron oxide) with morphologically and microstructurally distinct attributes and they preserve evidence of the microbial communities that thrived at that location when the sinter precipitated (i.e., primary communities) (Cady and Farmer, 1996; Renaut et al., 1998; Farmer and Des Marais, 1999; Fouke et al., 2000; Parenteau and Cady, 2010; Campbell et al., 2015). Once hydrothermal fluids vent at the surface, the dynamic combination of evaporation and cooling of thermal waters precipitates sinter deposits via heterogeneous and homogeneous nucleation and polymerization (Fournier and Rowe, 1977). Sinter precipitates begin to dry out along the hydrothermal terraces or cones as the distance to the spring vent increases and the hot fluids cool and evaporate. Sinter accretion typically encrusts, emtombs, and replaces these biological remnants (Cady and Farmer, 1996; Renaut et al., 1998; Campbell et al., 2015). The rapid, kinetically driven precipitation of mineraloids and minerals preserves organic biomarkers over geological time as distinct hydrothermal lithofacies (Schopf and Packer, 1987; Campbell et al., 2015; Westall et al., 2015). In addition, new microbial populations colonize (secondary communities) when the environmental conditions change along the hydrothermal activity lifetime. As a consequence, a variety of microbial communities thrive in distinct biofacies of geothermal springs. Hot spring deposits and the preservation of their biosignatures provide insights into the evolution of early life (Cady et al., 2018) and can inform astrobiological search strategies (Walter and Des Marais, 1993; Farmer and Des Marais, 1999).

The preservation of organic compounds in sinter deposits has been studied in geothermal springs of the major geyser fields worldwide, including Yellowstone National Park (*e.g.*, Jahnke et al., 2001; Pepe-Ranney et al., 2012), Iceland (*e.g.*, Konhauser et al., 2001; Tobler and Benning, 2011); Kamchatka (Goin and Cady, 2009), Tibet (Lau et al., 2008), or New Zealand (*e.g.*, Jones et al., 2001; Pantcost et al., 2005; Kaur et al., 2015). In comparison to most other hydrothermal settings, our understanding of organic preservation in sinter deposits at *El Tatio* (Chile), the third largest geyser field in the world and the largest in the southern hemisphere, is limited. Located within the Andes Mountains, *El Tatio* is one of the highest hydrothermal systems (4,320 mamsl), which subjects it to unique conditions such as intense UV-A and UV-B radiation, an unusually low water-boiling point (86°C), and severe climatic changes including large daily thermal oscillation and high atmospheric dryness (Fernandez-Turiel et al., 2005). These and other specific limiting factors for life at *El Tatio*, including a toxic chemistry of the geothermal-springs water (B, As, or Sb), make the geothermal field an extreme environment of interest for understanding the development and persistence of life under severe conditions, as well as a terrestrial model of a Martian environment. This model includes, apart from the extreme aridity, high solar radiation, salinity and oxidant conditions during the last 10–15 millions of years characteristic of the Atacama Desert environments (*e.g.*, Navarro-González et al., 2003), presence of volcanic and hydrothermal activity such as that in the ancient Mars. *El Tatio* serves as a natural laboratory for the study of the biogeochemical processes involved in the deposition and alteration of siliceous sinter and the potential for preserving microbial biosignatures. Yet, the few existing microbiological studies on *El Tatio* are focused on petrographic and mineralogical examinations (Fernandez-Turiel et al., 2005), thermal imaging (Dunckel et al., 2009), electron microscopy and UV-spectroscopy (Phoenix et al., 2006), or optical/scanning electron microscopy and molecular (DNA) methods (Barbieri et al., 2014). To the best of our knowledge, no studies have integrated microbiological and biogeochemical approaches for exploring the preservation of microbial biosignatures on sinter deposits from geothermal springs at *El Tatio*.

In this work, we investigated the presence and potential to preserve molecular biomarkers in sinter deposits from three geothermal-spring mounds at *El Tatio*. The springs are within 75 m of each other in the Upper Basin of *El Tatio*. Thermal features in the basin tap a ∼ 200–220 C hydrothermal reservoir at <250 m in the subsurface (Giggenbach, 1978; Muñoz-Saez et al., 2018). As in geyser basins worldwide, individual thermal features transition between actively emitting water or steam, and inactivity. This episodic nature of thermal activity is dictated by the subsurface fracture system, which is maintained through earthquake activity (Fournier, 1989). Consequently, any study of geysers and hot springs represents a snapshot in time. We use this snapshot to document and compare the microbial community and its associated biogeochemistry of each feature. The nature of the hydrothermal activity (or lack thereof) of these three mounds represented different stages in the lifetime of such features and ranged from abundant water discharged during geyser activity to steam to complete dryness. Because the system is dynamic, there is the potential for population shifts in the microbial communities as environmental conditions change (Smith et al., 2003; Lynne, 2015). This snapshot, therefore, combines the signatures of the original microbial community with those of any opportunistic communities that might arise as conditions change. We use visual and petrographic observations to control for such changes. We combined the use of lipid biomarkers and organic isotopic composition with immunological (sandwich microarray immunoassay) and genomic (DNA sequencing) techniques to investigate the microbial community and functionality in the three sinter mounds. Characterization of the morphological features and geochemistry of the sinter provided an ecological framework for interpreting the molecular and isotopic results. Compound-specific isotopic analysis of lipid biomarkers was employed to obtain information about different carbon cycling pathways; DNA sequencing was used to characterize the phylogenetic groups; and antibody microarrays were interrogated to demonstrate the presence of certain microbial strains and proteins involved in some metabolic and/or environmental traits. Mineralogy was identified with X-ray diffraction, and lithofacies traits preserved in the sinters were revealed by optical and electron microscopy. This multi-analytical (molecular, isotopic, genomic, mineralogical, geochemical, and paleobiological) approach was successful in explaining the influence of the degree of hydrothermal activity on the biomarkers record (i.e., from past and present) in sinter deposits from high altitude mounds at *El Tatio*. This is the first multidisciplinary molecular study of the biogeochemical evidence preserved in the sinter formations at *El Tatio*, which reveals more about the habitability, adaptability, and preservation of biosignatures in this type of Mars analog environment.

## Materials and Methods

### Field Settings

**FIGURE 1:**
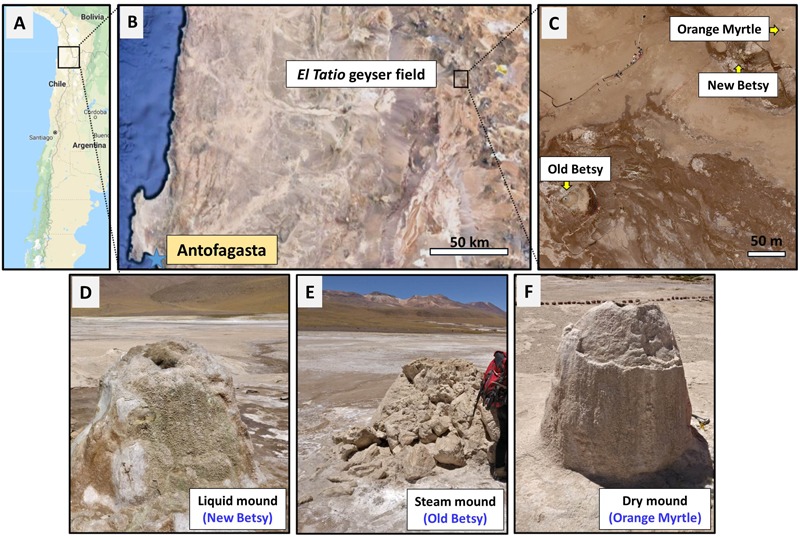
Site of study and sampling. Map of northern Chile **(A)**, showing the location of the *El Tatio* geysers field **(B)**, including the three geysers studied here (New Betsy, NB; Old Betsy, OB; and Orange Myrtle, OM) **(C)**. Both pictures in **B,C** are satellite images from Google Maps. The general appearance of the three sinter mounds (liquid NB, steam OB, and dry OM) is shown in **D–F**, respectively.

*El Tatio* geysers field (22°20′S and 68°W) is located in the Andean highlands (*i.e.*, Altiplano) near the Atacama Desert, northern Chile (Figure 1). This hydrothermal area consists of three distinct basins: Upper, Middle, and Lower Geyser Basins. The Middle Basin is composed of pools, fountain-type geysers, and the runoff streams from geothermal springs and pools (Glennon and Pfaff, 2003). The *El Tatio* geysers field, located along the Salado River Valley, contains more than 80 active geysers, fumaroles, geothermal springs, and mud volcanoes and is surrounded by extensive sinter terraces and aprons that spread over an area of approximately 10 km^2^. *El Tatio* is in a geological region composed of Jurassic marine sediments, Jurassic-Cretaceous andesites, Cretaceous sediments, Miocene ignimbrites and andesites, and Plio-Holocene lavas, domes, dacitic and rhyolitic ignimbrites (Lahsen and Trujillo, 1976). This geological sequence is overlain by glacial and alluvial deposits, which are locally covered by silica sinter deposits (Fernandez-Turiel et al., 2005). The extensive siliceous sinter formations at *El Tatio* are the result of silica precipitation from near-neutral thermal waters with a SiO_2_ concentration of 147–285 mg/l (Nicolau et al., 2014). Sedimentary microtextures in the sinter deposits suggested that the microbial community at *El Tatio* is moderately diverse, with a variety of extreme biological communities of thermophilic bacteria (*Chloroflexus*-like), cyanobacteria, and diatoms (Fernandez-Turiel et al., 2005).

### Sample Collection

Samples were collected from *El Tatio* geysers field (Figure 1) in October 2016, during a NASA Astrobiology Institute NAI-CAN 7 project (“Changing Planetary Environments and the Fingerprints of Life”) sampling campaign. Sinter samples were collected from three sinter mounds that appeared similar based on their size and the shape of the mounds, though differed in terms of their hydrological environment. As shown in Figure 1, the active-geyser mound known as New Betsy (NB) had abundant liquid water (∼84°C) that flowed from the mound, episodically covering the mound surface; the morphologically similar mound known as Old Betsy (OB) had a supply of steam (∼75°C) that enveloped various surfaces of the mound where samples were collected; and the inactive mound known as Orange Myrtle (OM) lacked both hydrothermal water and steam. For convenience, and to emphasize the distinct differences in the hydrological regime, we refer to the collected sinter samples as belonging to the liquid, steam, and dry mounds (Figures 1d–f). About 100 g of sinter sample were collected from equivalent sampling spots (i.e., half way down) from the three sinter mounds with a geological hammer and broken samples were gathered with a solvent-cleaned (DCM and MeOH) stainless-steel spatula. They were wrapped in aluminum foil and transported in solvent-clean containers for biogeochemical analysis at the CAB (*Centro de Astrobiología*). A sample of hydrothermal fluid was also collected from New Betsy for geochemical analysis. Physical splits of the samples were distributed to collaborators at different locations in the United States.

### Lithofacies and Scanning Electron Microscopy

The lithofacies of the sinter samples were described using terminology consistent with the lithofacies model for silica sinters provided by Walter (1976), based on the gross morphological features of laminated sinters. The sinter samples were air dried during transport and analyzed as bulk fragments to identify the distribution of morphological features consistent with silicified biological remnants in the context of their biofabrics. The biofabrics were identified with conventional stereoscopic and a scanning electron microscopic methods on fractured and sawn surfaces oriented perpendicular to the lamination. The use of the biological holder of a Phenom Pro scanning electron microscope (SEM) eliminated the need for carbon coating, though smaller fractured fragments of sinter were often carbon coated to reduce artifacts in the SEM images that were created by regions of higher porosity.

### Mineralogical and Geochemical Analyses

X-ray diffraction analysis was performed on different splits of the sinter samples at CAB and PNNL (Pacific Northwest National Laboratory). At CAB, the three sinter samples were analyzed using a Bruker X-Ray diffractometer (Eco-D8 advance, XRD) to determine the silica phase and associated mineralogy. Dry samples of the three sinter mounds were ground, mounted on a PMMA specimen holder and scanned between 5° to 60° 2𝜃, with a scanning step size of 1 s and 0.05°, operated at 40 kV and 25 mA with a Cu X-ray source (Cu Kα1,2, λ = 1.54060 Å). At PNNL, X-ray diffraction data were collected with the use of a Panalytical MPD Bragg-Brentano goniometer fitted with a Cu X-ray source operated at 45 kV and 40 mA, fitted with variable divergence slits (10 mm illuminated length) and a post-diffraction monochromator. The powders were loaded into the cavity of a zero-background holder and patterns were collected between 5 and 100° 2𝜃 with 4 s counts at 0.04° intervals.

Inorganic anions and organic acids of low molecular weight were determined by ion chromatography in the water-extractable phase of the three sinter samples (liquid, steam, and dry), or in the water sample from New Betsy, according to previous descriptions (Parro et al., 2011a; Sánchez-García et al., 2018). Briefly, 2 g of the sinter samples and 1 ml of the water sample were sonicated (3 min × 1 min cycles) and diluted in 10 mL of deionized water; then filtered (0.22 μm GFF), and analyzed in a Metrohm 861 Advanced compact ion chromatographer (Metrohm AG, Herisau, Switzerland), using 3.6 mM sodium carbonate (NaCO_3_) as eluent.

Stable isotopes of organic carbon (δ^13^C) and total nitrogen (δ^15^N) were measured on the bulk sinter samples with isotope-ratio mass spectrometry (IRMS), following USGS methods (Révész et al., 2012). Briefly, sinter samples (2 g) were homogenized by grinding with a corundum mortar and pestle. Subsequently, HCl was added to the samples to remove carbonates, equilibrated for 24 h, and adjusted to neutral pH with ultrapure water. The residue was then dried in an oven (50°C) for 72 h or until a constant weight was achieved and analyzed in the IRMS (MAT 253, Thermo Fisher Scientific). δ^13^C and δ^15^N values were reported in the standard per mil notation using three certified standards (USGS41, IAEA-600, and USGS40) with an analytical precision of 0.1‰. The content of total organic carbon (TOC %) and total nitrogen (TN %) was measured with an elemental analyzer (HT Flash, Thermo Fisher Scientific), during the stable isotope measurements.

### Geolipids Extraction, Fractionation, and Analysis

About 50 g of the sinter samples were Soxhlet extracted (24 h) with a mixture (ca. 250 ml) of dichloromethane/methanol (DCM/MeOH, 3:1, v/v), after addition of internal standards (tetracosane-D_50_, myristic acid-D_27_, 2-hexadecanol). The total lipid extracts were concentrated to ca. 2 ml by rotary evaporation and elemental sulfur removed overnight with activated copper. The clean extract was separated into two fractions of different polarity (neutral and acidic) using Bond-elute (bond phase NH_2_, 500 mg, 40 μm particle size) chromatography columns. A neutral lipid fraction was obtained by eluting with 15 ml DCM/2-propanol (2:1, v/v) and an acidic fraction with 15 ml of acetic acid (2%) in diethyl ether. Further separation of the neutral fraction into non-polar and polar sub-fractions was done with 0.5 g of alumina (activated, neutral, 0.05–0.15 mm particle size) in a pre-combusted Pasteur pipet. The non-polar fraction was obtained by eluting 4.5 ml of hexane/DCM (9:1, v/v) and the polar fraction with 3 ml of DCM/methanol (1:1, v/v). The acidic fraction was derivatized with BF_3_ in methanol and the polar fraction with N,O-bis (trimethylsilyl) trifluoroacetamide (BSTFA).

The three lipid fractions (non-polar, acid, and polar fraction) were analyzed with gas chromatography mass spectrometry using a 6850 GC system coupled to a 5975 VL MSD with a triple axis detector (Agilent Technologies) operating in conditions previously described elsewhere (Sánchez-García et al., 2018). For the non-polar fraction, the oven temperature was programmed from 50 to 130°C at 20°C min^-1^ and then to 300°C at 6°C min^-1^ (held 20 min); for the acidic fraction, from 70 to 130°C at 20°C min^-1^ and then to 300°C at 10°C min^-1^ (held 10 min); and, for the polar fraction, the oven temperature program was the same as that for the acidic fraction, except that the oven was held for 15 min at 300°C. The injector temperature was 290°C, the transfer line was at 300°C, and the MS source at 240°C. Compounds identification was based on the comparison of mass spectra with reference materials, and their quantification on the use of external calibration curves of *n*-alkanes (C_10_ to C_40_), fatty acids methyl esters (FAME; C_8_ to C_24_), *n*-alkanols (C_10_, C_14_, C_18_, and C_20_), and branched isoprenoids (2,6,10-trimethyl-docosane, crocetane, pristane, phytane, squalane, and squalene). All chemicals and standards were supplied by Sigma Aldrich. The recovery of the internal standards averaged 69 ± 18%.

### Compound Specific Isotope Analysis

Carbon isotopic compositions of individual lipid compounds (*n*-alkanes, carboxylic acids as FAMEs, and *n*-alkanols) were performed coupling the gas chromatograph (Trace GC 1310 ultra) to the isotope-ratio mass spectrometry system (MAT 253 IRMS, Thermo Fisher Scientific). The conditions for the GC analysis were identical to those used for the polar fraction analysis, whereas the conditions for the IRMS analysis were as follows: electron ionization 100 eV, Faraday cup collectors *m/z* 44, 45, and 46, and a temperature of the CuO/NiO combustion interface of 1000°C. The samples were injected in splitless mode, with inlet temperature of 250°C, and helium as a carrier gas at constant flow of 1.1 ml min^-1^. The isotopic values of the individual lipids separated by GC were calculated using CO_2_-spikes of known isotopic composition, introduced directly into the MS source, three times at the beginning and end of every run. Reference mixtures (Indiana University, United States) of known isotopic composition of *n*-alkanes (A6) and FAMEs (F8) were run after every four samples to check accuracy of the isotopic ratio determined by the GC-IRMS. The δ^13^C data for individual carboxylic acids (*i.e., n*-carboxylic acids, *iso*/*anteiso* and unsaturated) were calculated from the obtained FAME values, by correcting them for the one carbon atom added in the methanolysis (Abrajano et al., 1994).

### DNA Extraction, PCR Amplification, and DNA Sequencing

Genomic DNA was extracted from the three sinter samples, using the CTAB genomic DNA extraction method (Warren-Rhodes et al., 2018). Bacterial 16S rDNA V3-V4 gene region from all DNA extracts was then PCR amplified, using the primer pairs 341-F/805-R (Herlemann et al., 2011). Archaeal 16S rDNA V3-V4 region was PCR amplified using the primer pair Arch1F/Arch1R (Cruaud et al., 2014) only from the dry extract, due to the little amount of archaeal biomass in the liquid and steam samples. Both bacterial and archaeal PCR amplifications were carried out according to standard procedures (Warren-Rhodes et al., 2018). Microbial communities were then identified by the construction of a paired-end amplicon library by means of Illumina MiSeq sequencing (Illumina Inc., San Diego, CA, United States). Raw sequence data were deposited at the NCBI Sequence Read Archive (SRA^1^), under accession number PRJNA507699.

Raw sequences were processed either in the MOTHUR software v.1.39.5 (Schloss et al., 2009), using a custom script based upon MiSeq SOP (Kozich et al., 2013), or in R package ‘phyloseq’ (McMurdie and Holmes, 2013). Sequence reads were clustered into OTUs (Operational Taxonomic Units) at the 97% similarity level. Datasets were rarefied independently by random selection to even sequencing depth, corresponding to the lesser number of sequences found in the samples (*i.e.*, 40264 reads). Taxonomic affinities for the reads were assigned by comparison of OTUs representative sequences against RDP database (RDP reference files v.16; release 11, Cole et al., 2014). OTU’s affinities reported as “cyanobacteria/chloroplast” were further assigned to a taxonomic identity by comparing them against nr/nt (NBCI), EMBL, Greengenes and SILVA databases for more precise cyanobacteria taxonomic identification. The sequences assigned to “mitochondria” or “chloroplast” were removed from further analyses. The total number of OTUs was provided as an estimate of phylogenetic richness. Shannon’s diversity index (*H’*) and Pielou’s Evenness (*J’*) based on OTU data were calculated on the three samples by means of R package ‘vegan’ (Oksanen et al., 2017). The same package was also used to perform a Correspondence Analysis (CA) between microbial classes and sinter samples.

### Multiplex Fluorescent Sandwich Microarray Immunoassay (FSMI)

Powdered sinter samples were analyzed by fluorescent sandwich microarray immunoassays (FSMI) with the LDChip200 (*i.e.*, Life Detector Chip; Parro et al., 2008a, 2011a), to interrogate a panel of about 200 polyclonal antibodies produced for binding with biological polymers and microbes from extant or well-preserved extinct life structures (Rivas et al., 2008). The LDChip200 used in this study contained 181 antibodies (purified IgG fraction) produced using as immunogens: (i) whole microbial cells from bacteria and archaea, (ii) spores from Gram-positive bacteria, (iii) extracellular polymeric substances from cultures and environmental samples, (iv) environmental extracts (from soils, water, sediments, rocks, and biofilms) from extreme environments (Parro et al., 2011a; Blanco et al., 2017), (v) conserved proteins and peptides involved in key metabolisms (*e.g.*, nitrogen fixation, nitrogen and sulfur reduction, energy metabolisms, iron storage, or PHAs (poly-hydroxyalkanoates) synthesis), and (vi) 36 preimmune sera (IgG fraction) as negative controls (for a detailed antibody information see Supplementary Table S1). The targets for the 181 antibodies used in this work are described elsewhere (Supplementary Table S1 in Sánchez-García et al., 2018) and complemented with the antibodies listed in Supplementary Table S1 in this work. The purified immunoglobulin (IgG) fraction of each antibody was printed in a triplicate spot-pattern, fluorescently labeled with Alexa 647, checked, titrated and used as reported elsewhere (Rivas et al., 2008).

The LDChip200 is a shotgun antibody microarray immunosensor produced to increase the success of detecting any microbial remain in natural samples, either for environmental monitoring and/or for detecting signs of life in planetary exploration (Rivas et al., 2008; Parro et al., 2008b, 2011b, 2018). Limitations related to the presence of relatively complex molecules from abiotic origin in other planetary bodies, are being presently achieved by adding new antibodies for detecting molecules such as aromatic amino acids or polyaromatic hydrocarbons (Moreno-Paz et al., 2018). In continuous process of improvement, the LDChip is the core sensor of the already high TRL (Technology Readiness Level) instrument called SOLID (*Signs of Life Detector*), specially conceived for missions concept as the IceBreaker drilling of the Martian permafrost (McKay et al., 2013). The detailed protocol for the analysis of the sinter samples at *El Tatio* with the LDChip200 is described in Blanco et al. (2017). Briefly, up to 0.5 g of each sample were resuspended in 2 mL of TBSTRR buffer (0.4 M Tris–HCl pH 8, 0.3 M NaCl, 0.1% Tween 20), ultrasonicated and filtered through 5 μm. The filtrates were used as a multianalyte-containing sample for the FSMI as described in preceding works (Rivas et al., 2008; Blanco et al., 2012, 2017). The LDChip200 microarray images were analyzed and quantified by GenePix Pro Software (Molecular Devices, Sunnyvale, CA, United States). The final fluorescence intensity (*F*) of each antibody spot was calculated as reported by Rivas et al. (2011). To minimize the probability of false positives, we increased the stringency by applying to all spots an additional cutoff value of 2.5-fold the average of *F* of the whole array (Blanco et al., 2015; see details on Supplementary Text S1).

In addition, the molecular abundance, richness, diversity, and evenness were also estimated. As all the immunoassays were performed on the same amount of sample and upon similar experimental and scanning conditions, the sum of fluorescence intensity in every positive antigen-antibody reaction for each sample was used as an estimation of the molecular abundance (Parro et al., 2011c), whereas the number of positive antigen-antibody reactions was employed as an indirect measure of the sample richness. The molecular diversity was then determined through the Shannon Index (*H’*), according to the following equation:

(1) $$H\text{′}=-\sum_{i=1}^{s}\left(p_{i}\log_{2}p_{i}\right)$$

where *H’* defines the molecular diversity in the samples analyzed by the LDChip200; *s* corresponds to the molecular richness (*i.e.*, sample richness above) and *p_*i*_* is the partial diversity calculated as a rate between the fluorescence for each positive immuno-detection and the total fluorescent for each sample under analysis. Finally, the molecular evenness at each sample was then determined through the Pielou’s evenness index (*J’*), according to the following equation:

(2) J′=H′ / lnS

where *J’* defines Pielou’s evenness index; *H’* corresponds to the molecular diversity in the samples analyzed by the LDChip200; and S matches to the molecular richness.

## Results

### Silica Sinter Lithofacies in the Three Sinter Mounds at *El Tatio*

Stereoscope and SEM images of the liquid mound and steam mound sinter samples in different orientations illustrated they consisted primarily of spicules characterized by parabolic laminations of vitreous clear or opaque opal-A. Supplementary Figure S1A shows a cross-sectional stereoscopic view of closely spaced spicules in the liquid mound sample. The inner core of the spicules comprised dense vitreous opal-A, which did not always appear laminated on fractured surfaces of the structures. The outermost opaque and tan laminations of the spicules, which contrasted with the clear inner core of the structures, revealed their downward parabolic orientation that was visible due to differences in their color. The more porous and bright white precipitate located between the spicules was the remnants of silicified microbial biofilms. SEM images of the top surface of the liquid mound sample revealed the presence of heavily silicified and intertwined filaments (Supplementary Figure S1B) and less-silicified remnants of short filaments and rods on the most recent accretionary surface of the sample (Supplementary Figure S1C). SEM images from the inside of this sample illustrated entombment of microbial remnants in opal-A nanocolloids (Supplementary Figure S1D) and the remnants of exopolysaccharides (EPS) that are often preserved with a honeycomb-like structure on surfaces where microorganisms attached reversibly (leaving only EPS remnants) and irreversibly (leaving intertwined cellular remnants and EPS relicts) on exposed sinter surfaces (Supplementary Figure S1E).

Supplementary Figure S2A showed a top-down stereoscopic view of closely spaced spicules in the steam mound sample that were surrounded by the remnants of silicified biofilms and detrital grains. The downward parabolic laminations of the spicules were observed in this orientation as concentric laminae, once again distinguishable as variations in color (white, tan, and gray). A SEM image of the abrupt interface between the highly porous silicified biofilm and massive vitreous opal-A (Supplementary Figure S2B) suggested that once environmental conditions supported biofilm growth on the periphery of a spicule, that growth continued unabated until the structure was intermittently immersed in hydrothermal fluid, allowing spicular growth to resume. The pore space between closely spaced spicules in the steam sinter was completely filled with a combination of silicified biofilm and detrital grains of opal-A and accessory minerals. A SEM image of a spicule separated from the sample (Supplementary Figure S2C) showed relicts of silicified biofilm attached to the spicule surface and, occasionally, cellular remnants of bacteria that either colonized the spicular surfaces (Supplementary Figure S2D) or infiltrated the pore space of previously silicified remnants (Supplementary Figure S2E).

Supplementary Figure S3A showed a cross-sectional stereoscopic view of wavy laminations of opal-A in the sinter sample from the dry mound. A stereoscopic plan view of the dry mound sample (Supplementary Figure S3B) showed evidence of an irregular network of ridges. The wavy lamination visible in cross-section (Supplementary Figure S3A) is due to the ridge-like nature of the accretionary surface. An SEM image of this surface showed the well-preserved silicified remnants of a biofilm that consisted primarily of short filaments, rods, and the fibrils typical of dehydrated EPS (Supplementary Figure S3C). Heavily entombed filaments (Supplementary Figure S3D) and organically preserved remnants of a biofilm that consisted of cocci (Supplementary Figure S3E) were also found on the surfaces and in fractures of the dry sinter sample.

### Mineralogy and Bulk Geochemistry of the Three *El Tatio* Sinter Mounds

The X-ray diffraction patterns produced by fragments of spicules and columns that were separated from the liquid and steam mound samples and from a non-porous, white region from the top of the dry sinter (Supplementary Figure S4) revealed that they consisted almost entirely of opal-A (Rodgers et al., 2004). As an aqueous precipitate, the mineraloid opal-A does not have a three-dimensional crystalline structure, yet the ubiquitous presence of randomly ordered silica tetrahedra produces a diffraction pattern that consists of a single broad feature centered on 23° 2𝜃 (ca. 3.9 Å), typical of X-ray amorphous materials. The matrix of the liquid and steam mounds consisted of fine-grained detrital material primarily formed from opal-A (Supplementary Figure S4) and the accessory phases quartz, halite, feldspar, clay minerals, and iron oxides (data from analysis at CAB, not shown). Distinct detrital layers with a similar accessory mineral inventory were also found in the dry mound sinter sample.

**FIGURE 2:**
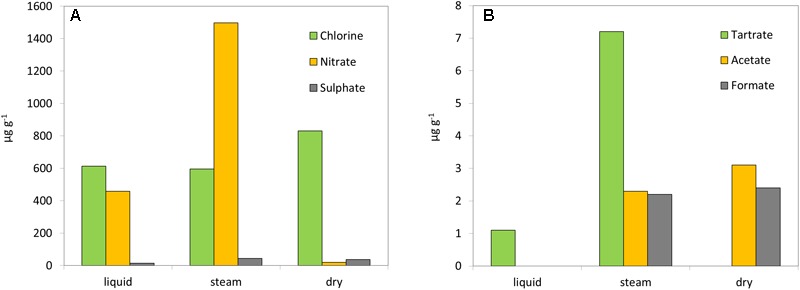
Geochemical analysis. Concentration (μg g^-1^) of inorganic **(A)** and organic anions **(B)** in the three sinter mounds at *El Tatio* (liquid, steam, and dry).

Ion chromatography (IC) showed the presence of diverse inorganic anions (Figure 2A), in the three sinter samples with variable content depending on the hydrothermal system (*i.e.*, liquid, steam, or dry). Chloride was present in the three sinter extracts, with similarly high concentrations in the liquid (612 μg g^-1^) and steam (596 μg g^-1^) mound samples, and an even higher concentration in the dry one (831 μg g^-1^). Nitrate was present in the three systems, with the steam sample containing the largest concentration of all mounds and ions (1497 μg g^-1^) and the dry sample the lowest (20 μg g^-1^). In fact, nitrate and chloride concentrations were inversely related among the three mounds. Sulfate was measured in low concentrations (<45 μg g^-1^) in all samples, as well as fluoride (<20 μg g^-1^) (Supplementary Table S2). The water collected from the liquid mound contained higher concentrations of chloride (7068 μg g^-1^), sulfate (346 μg g^-1^), fluoride (12 μg g^-1^), and bromide (56 μg g^-1^) compared to its sinter deposits (Supplementary Table S2).

In addition to the inorganic anions, three light organic acids were detected by IC (Figure 2B). Acetate and formate were present at similarly low concentrations (<2.5 μg g^-1^) in the extracts from the steam and dry sinter samples, but not detected in that of the liquid mound sample. Conversely, extracted tartrate was measured at higher concentration in the steam (7.2 μg g^-1^) than the liquid (1.1 μg g^-1^) samples, but was not detected in the dry sample. The water sample from the liquid mound was measured to contain only tartrate, which was present in very high concentration (286 μg ml^-1^) (Supplementary Table S2).

The content of TOC (0.07–0.10%) and TN (0.01–0.04%) varied little between the three sinter samples (Table 1), with the dry mound showing the lowest values of both elements. The resulting TOC over TN ratios (C/N) varied from 3 to 7. The biomass isotopic ratios δ^13^C and δ^15^N ranged from -15.7 to -24.0‰ and from -0.9 to 5.4‰, respectively (Table 1). The steam sinter sample showed the most depleted (δ^13^C) and enriched (δ^15^N) isotopic composition.

**Table 1:** Bulk geochemical composition of the sinter samples from the three mounds at *El Tatio*.

	Liquid	Steam	Dry
TOC (% dw)	0.10	0.10	0.07
TN (% dw)	0.02	0.04	0.01
δ^13^C OC (‰)	-15.9	-24.0	-15.7
δ^15^N TN (‰)	-0.9	5.4	-1.1
C/N	5	3	7
*n*-alkanes	0.05	0.08	0.05
Branched alkanes^a^	0.11	0.14	0.04
Octadecene (C_18:1_)	n.d.	0.04	n.d.
Hentriacontatriene (C_31:3_)	n.d.	0.01	n.d.
*n*-carboxylic acids	2.53	2.71	2.35
Unsaturated carboxylic acids^b^	0.39	0.34	0.23
Dicarboxylic acids^c^	0.06	0.05	0.03
*Iso-/anteiso* carboxylic acids^d^	0.71	0.38	0.24
Other branched carboxylic acids^e^	0.11	0.02	0.01
Cyclopropyl acids^f^	n.d.	n.d.	0.03
*n*-alkanols	1.40	1.12	1.81
Stigmastanol	0.12	0.13	0.06
β-sitosterol	0.01	0.04	n.d.
Cholesterol	n.d.	0.01	0.17
Pristane	0.05	0.12	n.d.
Phytane	0.21	0.35	n.d.
Squalane	n.d.	n.d.	0.003
Crocetane	n.d.	n.d.	0.002

*Concentration (μg g^-*1*^) and compositional distribution of lipid biomarkers. ^*a*^Sum of mono-methyl, di-methyl and tri-methyl alkanes. ^*b*^Sum of mono- and di-unsaturated carboxylic acids between 16 and 19 carbon units. ^*c*^Sum of dicarboxylic acids between 6 and 10 carbon units. ^*d*^Sum of *iso* and *anteiso* carboxylic acids between 15 and 19 carbon units. ^*e*^Sum of other mono-methyl (MM) carboxylic acids. ^*f*^Sum of cyclopropyl C_*17*_ and C_*19*_ acids. Please go to Supplementary Figures S5–S8 for consulting individual concentrations of the (*normal*, branched, and other) carboxylic acids, alkanols, and alkanes families.*

### Molecular Distribution of Lipid Biomarkers in the Three Sinter Mounds

**FIGURE 3:**
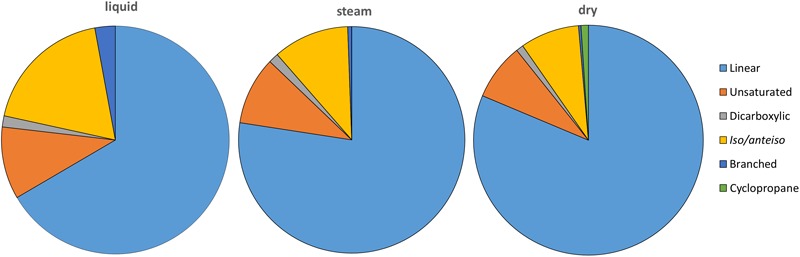
Relative amount (%) of carboxylic acids (linear and saturated, or *normal*; unsaturated; dicarboxylic; *iso/anteiso*; other branched; and cyclopropyl) in the three sinter mounds at *El Tatio*. “Unsaturated” includes mono- and di-unsaturated carboxylic acids, whereas “other branched” covers middle chain mono-methyl carboxylic acids.

Several lipid families were detected in the three sinter samples, with a generalized larger abundance of the functionalized lipid groups. The most abundant class of lipids were the linear carboxylic acids (*i.e., n*-carboxylic acids), which concentration ranged from 2.35 to 2.71 μg g^-1^ (Table 1). The molecular distribution of the *n*-carboxylic acids showed chain lengths ranging from C_8_ to C_30_, a clear dominance of the even carbons, maximum at C_16_ or C_18_, and secondary groups at C_22_ to C_26_ (Supplementary Figure S5). After the straight-chained congeners, the most abundant carboxylic acids were those with branches at *iso-* and/or *anteiso-* positions (0.24–0.71 μg g^-1^), followed by the unsaturated (0.23–0.39 μg g^-1^), dicarboxylic (0.03–0.06 μg g^-1^), and cyclopropyl (0.03 μg g^-1^) congeners (Figure 3). *Iso* and *anteiso* carboxylic acids of 13 to 19 carbon units were found in the three sinter samples (Supplementary Figure S6), with more of the *iso/anteiso*-C_18_ in the liquid and dry mounds. Carboxylic acids with one or two unsaturations were generally measured at chain lengths of C_16_ (16:1 ω7, and 16:1 ω9), C_17_ (17:1 ω6), C_18_ (18:1 ω9, and 18:2 ω6,9), and C_19_ (19:1 ω9) (Supplementary Figures S6D–F). Dicarboxylic acids of short chain (C_6_ to C_10_) and branched carboxylic acids other than *iso/anteiso* congeners (*i.e.*, monomethyl acids, MM) were found to be minority congeners in the acidic fractions (Figure 3). Cyclopropyl C_17_ and C_19_ acids were only detected in the dry sinter sample at a total concentration of 0.03 μg g^-1^ (Table 1).

Other functionalized lipids, the straight chain alkanols (*i.e., n*-alkanols), were found at chain lengths between C_10_ and C_29_, and concentrations ranging from 1.12 to 1.81 μg g^-1^ (Table 1). Similarly to the *n*-carboxylic acids, the molecular distribution of the *n*-alkanols showed a markedly even character dominated by the C_16_ and C_18_ congeners (Supplementary Figure S7). Together with the linear alkanols, two phytosteroids (β-sitosterol and stigmastanol), and cholesterol were identified within the polar fraction (Table 1). The largest amount of cholesterol was measured in the sinter sample from the inactive, dry mound.

In contrast to the functionalized lipids, saturated hydrocarbons were less abundant in the three sinter samples (Table 1). Branched alkanes including mono-, di-, and tri-methyl ramifications were detected at one order of magnitude larger concentration (0.11–0.14 μg g^-1^) than the *n*-alkanes (0.05–0.08 μg g^-1^) in the liquid and steam sinter samples. In the dry mound, the concentration of branched and *n*-alkanes was similar. The *n*-alkanes distribution ranged in the three sinter samples from C_10_ to C_33_, and showed different maximum peaks at C_17_ (liquid mound), C_15_ (steam mound), or C_25_ (dry mound) (Supplementary Figures S8A–C). Among the branched alkanes, the mono-methyl congeners (C_15_, C_17_, and C_18_) were the most abundant in all sinter samples (Supplementary Figures S8D–F), especially in the steam mound. Other branched alkanes of isoprenoid configuration were detected at variable concentrations among the sinter samples. Pristane and phytane were present in the liquid and steam mounds, whereas the dry sinter contained only small traces of squalane and crocetane (Table 1). Low concentrations of octadecene (C_18:1_) and hentriacontatriene (C_31:3_) were also found in the steam sinter sample (Table 1).

### Isotopic Distribution (δ^13^C) of Lipid Biomarkers in the Three Sinter Mounds

**FIGURE 4:**
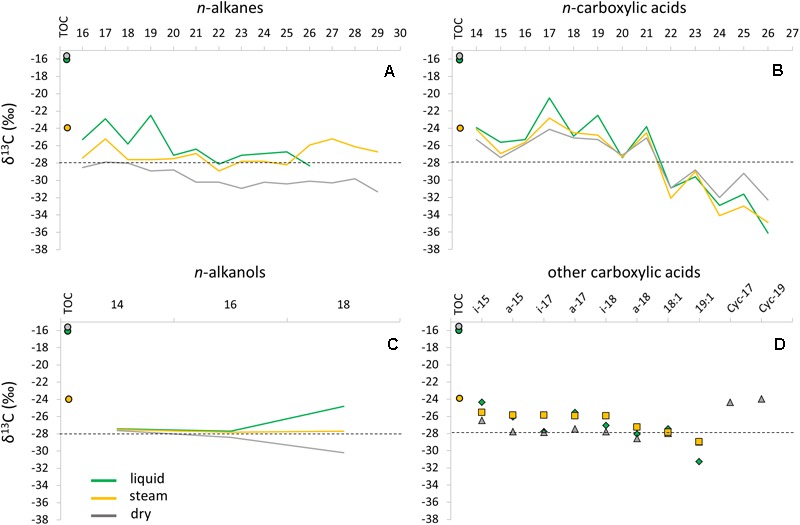
Compound-specific isotopic composition (δ^13^C) of specific lipid families in the three sinter mounds (liquid, steam, and dry); *n*-alkanes **(A)**, *n-*carboxylic acids **(B)**, *n-*alkanols **(C)**, and *iso*/*anteiso-*, unsaturated, and cyclopropyl (Cyc 17:0 and Cyc 19:0) acids **(D)**. The bulk isotopic ratio of the total biomass (*i.e.*, relative to TOC) was also depicted for the three samples as full circles. A dashed line was provided in each panel to facilitate the visualization of the δ^13^C shifts observed (mostly) in the *n*-carboxylic acids.

The compound-specific carbon isotopic ratio of alkanes, carboxylic acids and alkanols showed certain trends between the samples (Figure 4). The *n*-alkanes δ^13^C values ranged from -22.5 to -31.3‰ in the three sinter samples, with a general depletion with increasing number of carbon units, and from the liquid to the dry mound (Figure 4A). In the liquid sinter sample, a clear enrichment was observed for the C_17_ (-22.9‰) and C_19_ (-22.5‰) relative to the remaining *n*-alkanes. The δ^13^C composition of the *n*-carboxylic acids ranged from -20.5 to -36.1‰ (Figure 4B), with an even clearer depletion with increasing carbon units and a marked enrichment of the odd relative to the even carbons. The compound-specific isotopic ratio of other acids could only be achieved for a few *iso*/*anteiso*, unsaturated, and cyclopropyl congeners (Supplementary Table S3). The *iso/anteiso* and unsaturated acids contained generally homogeneous δ^13^C ratios, whereas the cyclopropyl C_17_ and C_19_ acids only found in the dry mound were relatively enriched (Figure 4D). Only three members of the *n*-alkanols family (C_14_, C_16_, and C_18_) were detectable for their ^13^C content (Supplementary Table S3). The three samples showed similar δ^13^C values for the C_14_ (-27.4 to -27.6‰) and C_16_ (-27.7 to -28.4‰) *n*-alkanols, whereas a distinct composition was observed for the C_18_ congener in the liquid (-24.8‰), steam (-27.7‰), and dry (-30.2‰) samples (Figure 4C). Overall, all lipid groups showed a depleting trend in the ^13^C composition from the liquid to the dry mound.

### Microbial Diversity Based on DNA Analysis

**FIGURE 5:**
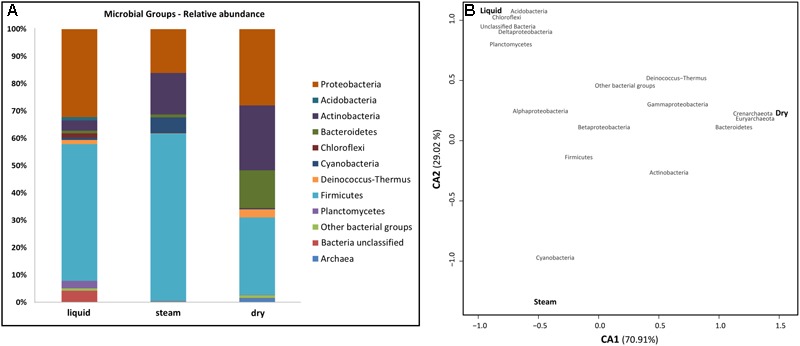
Comparison of microbial groups in the sinter samples from the three mounds at *El Tatio* **(A)**, and Correspondence Analysis (CA) between the main microbial groups (red) and the sampling sites (black) **(B)**. In a, the microbial groups inferred from DNA analysis are expressed as relative abundance. Bacillales and Clostridiales (Firmicutes), Rhodobacteriales, Rhizobiales, Burkholderiales and Desulfuromonadales (Proteobacteria), as well as Actinomycetales (Actinobacteria) were the most represented orders within the most abundant bacterial phyla. In b, CA1 (X axis) accounted for 70.90% of total variability explained by the model, and CA2 (Y axis) for 29.02%. The closer clustering of microbial groups to a sampling site is, the more characteristic these groups are at the site. Distance among sampling sites depicts compositional differences in them.

Phylogenetic analysis of environmental 16S rRNA gene amplicon sequences showed that almost 70% of total bacterial reads in the *El Tatio* sinter geysers belonged to the Firmicutes and Proteobacteria phyla (Figure 5A). The low GC Gram-positive Firmicutes were mainly found in the liquid and steam sinter samples, which showed a closer correspondence than the dry sample to that phylum (Figure 5B). Bacillales (Bacilli) and Clostridiales (Clostridia), together accounting for more than 80% of the Firmicutes’ sequences, were the most frequently represented orders. Proteobacterial classes were generally more abundant in the liquid mound, especially Alphaproteobacteria (17.6% in liquid mound versus 9.2 and 2.5% in steam and dry mounds, respectively). In contrast, Gammaproteobacteria were more represented in the dry (22% of total sequences) than in the liquid (9.9%) and steam (3.9%) mounds, as the correspondence analysis illustrated (Figure 5B). The orders Rhodobacteriales and Rhizobiales (Alphaproteobacteria), Burkholderiales and Hydrogenophilales (Betaproteobacteria), Desulfuromonadales (Deltaproteobacteria), as well as Vibrionales and Xanthomonadales (Gammaproteobacteria) together accounted for 70% of the total proteobacterial sequences.

The Actinobacteria (mainly Actinomycetales), Bacteroidetes (Cytophagales and Flavobacteriales), and Cyanobacteria together accounted for the 22% of the total number of sequences found in the sinter samples (Figure 5A). Actinobacteria and Bacteroidetes were particularly abundant in the dry mound (23.7 and 13.9% of sample sequences, respectively), whereas Cyanobacteria was more prevalent (5.9%) in the steam mound sample (associations illustrated in Figure 5B). In contrast, Acidobacteria, Chloroflexi, and Planctomycetes each accounted for >1% of the sequences only in the liquid sinter (Figure 5B). The Deinococcus-Thermus class is of special interest as it was observed to be similarly representative of both the liquid (1.7% of total sequences) and dry (2.9%) mounds (Figure 5B), with sequences only belonging to Thermales and Deinococcales orders, respectively.

Archaeal sequences were recovered only in the dry sample (Figure 5A). They accounted for <2% of overall diversity in these samples and comprised taxa from just two classes: Halobacteria (Euryarchaeota) and Thermoprotei (Crenarchaeota) (Figure 5B).

**FIGURE 6:**
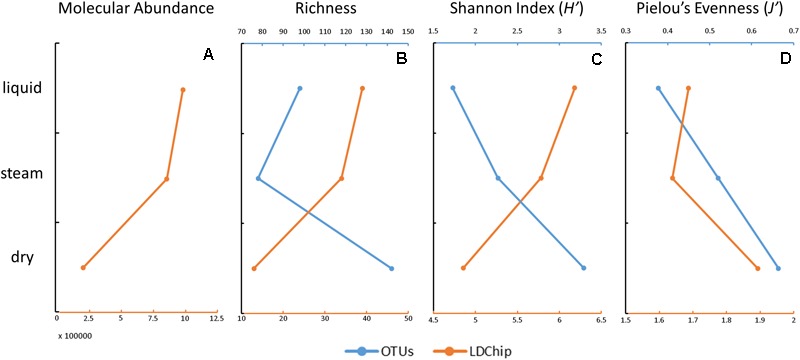
Estimation of the molecular abundance, richness, diversity, and evenness in the three sinter samples at *El Tatio*, based on the DNA sequencing (blue) and LDChip200 (orange) results. Molecular abundance **(A)** was only considered for the LDChip technique, as the sum of the fluorescence intensity in all positive immuno-detections in the LDChip200. Richness **(B)** was based on the total number of OTUs (DNA sequencing) and on the number of positive immuno-detections (LDChip200). The diversity **(C)** was based on the Shannon index in both cases. Community evenness **(D)** was based on Pielou’s evenness index. Please note that the opposite trend of richness and diversity in the LDChip200 and OTUs estimates were attributed to the different approach employed by both techniques (*i.e.*, close versus open methods, respectively).

The OTU-richness varied from 78 in the steam sample to 142 in the dry sample (Figure 6B). The Shannon diversity index (*H’*) of the microbial communities at OTU level showed increasing values from the liquid (1.7) to the steam (2.3) and dry (3.3) samples (Figure 6C), concurrently with the Pielou’s Evenness index (*J’* of 0.38, 0.52, and 0.67, respectively) based on the OTU data (Figure 6D).

### Microbial Mass and Biomarkers Detected by a Multiplex Immunoassay

**FIGURE 7:**
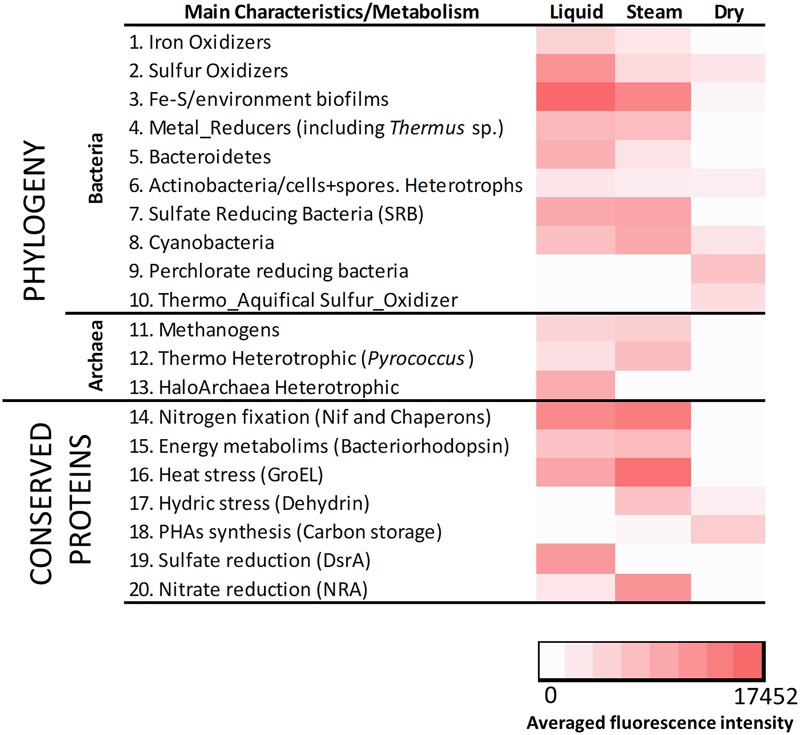
Heat map showing the results obtained with LDChip200 immunoassays. The antibodies (see Supplementary Table S3) were reorganized on the basis of main phylogenetic groups and metabolic traits of the target immunogens and protein functions in 19 different categories. The averaged fluorescence intensity of those positive immuno-detections within the same category (Supplementary Figure S9), obtained from three replicates per sample (that means nine spots per antibody), is plotted in a color scale from white (negative results) to red (maximum of 17452). Please note that 0 stands for those values under the limit of detection.

The LDChip200 revealed the presence in the sinter samples of biomolecules recognized by antibodies produced against iron and sulfur oxidizing bacteria and crude environmental extracts from biofilms of the Río Tinto area (Supplementary Figure S9) such as *Leptospirillum ferrooxidans, Acidithiobacillus* spp. and others (Amils et al., 2002). The immunoassays also detected positive immuno-signals against metal, sulfate and perchlorate reducers, bacteroidetes, actinobacteria including spore-forming bacteria, cyanobacteria, the thermo-aquifical sulfur oxidizer *Hydrogenobacter thermophilus* as well as methanogenic, thermophilic and halophilic archaea. In addition, positive immuno-detections were revealed with antibodies to proteins related to nitrogen fixation, energy metabolisms, thermal and hydric stress, poly-hydroxyalkanoates (PHAs) synthesis and sulfate and nitrate reduction (Supplementary Figure S9). Detection of chemolithoautotrophs involved in the use of sulfur and iron (Figure 7 and Supplementary Figure S9) revealed the occurrence of sulfur-iron metabolism, where sulfur and iron oxidizers could proliferate in oxygen-rich microniches, while metal (mainly iron) reducers occupied anaerobic microregions with organic matter available for oxidizing in parallel to the metal reduction.

A heat map showing the fluorescence intensity of the positive immuno-detections (Figure 7) displayed relative differences in the composition of the microbial community in the three sinter mounds. Chemolithoautotrophs involved in the oxidation of sulfur (*Acidithiobacillus* spp.), and oxidation (*Leptospirillum* sp. and *Acidimicrobium* sp.) or reduction of iron (*Acidocella* sp., *Acidiphilium* sp., and *Shewanella* spp.) were more abundant in the liquid and steam samples. These sinter samples (mostly the steam one) were also richer than the dry sample in primary producers such as benthic (*Anabaena* sp., *Leptolyngbya* sp., and *Tolypothrix* sp.) and endolithic (*Chroococcidiopsis* sp.) cyanobacteria, consistent with the higher detection of nitrogen fixation proteins (*i.e.*, Nif and chaperon HscA; Figure 7). Sulfate reducers were also detected in higher intensity in the steam and (mostly) liquid samples, through antibodies to *Desulfovibrio* sp. and *Desulfosporosinus* sp., as well as to the DsrA protein. In addition, positive immuno-detections of archaeal strains such as *Methanobacterium* sp., *Pyrococcus* sp. or *Halorubrum* sp., accounting for methanogenic and heterotrophic metabolisms, were only observed in the liquid and steam samples. In contrast, the dry sinter recorded the highest immuno-signals against (i) bacteria capable of perchlorate reduction (*Magnetospirillum* sp., *Ideonella* sp. and *Dechlorobacter* sp.), (ii) an aquifical bacterium (*Hydrogenobacter thermophilus*), and (iii) proteins related to the synthesis of PHAs (Figure 7).

The sum of fluorescence intensity decreased from the liquid to the dry mound by a factor of ca. 5 (Figure 6A), indicating that the dry sample contained lower biomass or that target microbial markers were transformed or degraded with time. The declining number of positive immuno-signals from the liquid (*n* = 39) to the dry (*n* = 13) sample resulted in parallel decreasing trends of the molecular richness (Figure 6B) and diversity (Figure 6C) toward the less active mound. In contrast, the Pielou’s evenness showed a somewhat increasing trend with the loss of hydrothermal activity (Figure 6D).

## Discussion

### Mineralogical, Geochemical, and Morphological Features in the Three Sinter Mounds at *El Tatio*

The mineralogical composition of the sinter samples was similar in the three geyser mounds, with amorphous silica phases (*i.e.*, opal-A) dominating the three XRD spectra (Supplementary Figure S4). The shift of the strongest feature (3.9 Å) to slightly higher d-spacing (3.93 Å) for the dry sinter relative to the liquid and steam sinters suggested a more dense packing of SiO_4_ tetrahedra in the dry sinter, consistent with dehydration. In the liquid and steam mounds, the sinter deposit was composed of spicular or columnar geyserite, which typically forms under conditions of intermittent inundation and splashing at temperatures >80°C (Walter, 1976; Cady and Farmer, 1996; Braunstein and Lowe, 2001). Evaporative salts and accessory minerals contribute to the tan and gray color of the matrix. The accessory mineral inventory is most likely wind-blown material from the volcanic country rock that surrounds the hydrothermal basin (Nicolau et al., 2014). Differences in the amounts of such detrital phases incorporated during accretion, often give these layers the appearance of being laminated. Yet these interlayers have an important role in defining the environmental conditions that define the habitat in each mound.

A geochemical difference among the mounds complements the mineralogical observations. The driest mound would be expected to have the most evaporative salt and, indeed, a higher concentration of chloride was found in the dry mound than in the other two samples (Figure 2A). The progressive wetting-drying cycles that would occur during waning geyser activity would likely concentrate salts evaporatively from the chloride-rich hydrothermal fluid. The biofabric of the dry sinter sample was consistent with this interpretation of prolonged periods of dryness at this inactive mound. During periods when the geyser mound was active, the relatively flat-to-wavy laminated sinter fabric would have developed when accretionary surfaces were perpetually wet and immersed in liquid for long periods of time. Continuous inundation would preclude spicule formation, which occurs on splashy, intermittently dry surfaces. A comparison of the random network of ridges on the outer surface of the dry sinter (Supplementary Figure S3) developed during the waning stages of geyser activity. That is, spicules develop in evaporative settings, ridges develop when there is intermittent wetting, and flat and wavy laminations develop when flow is continuous. These three environments are visible in the organic compositions and microbial populations as well.

The morphological characteristics of the biofabrics of the three sinter samples confirmed that the liquid and steam mounds displayed gross morphological and biofabric evidence of having formed in an intermittently wetted environment. Under such conditions, fluid is typically drawn by capillary action to topographical highs on the sinter due to rapid evaporation at the apices of spicular protrusions (Cady and Farmer, 1996). Walter (1976) was the first to describe spicular and columnar geyserites formed along the rims and edges of hot springs in Yellowstone National Park (United States) and he attributed their morphology to episodic splashing at the nearby pool. At *El Tatio*, spicular geyserite structures occurred on the tops and sides of the liquid and steam mounds in distinct regions that appeared to be associated with either (i) intermittent fluid flow from the top of the erupting liquid geyser mound or (ii) from a region within the base of the steam mound that was engulfed in steam from hot fluid inside the sinter cone at the base of the structure. Regardless of the distribution of these structures on the top, side, or base of the mounds, the liquid and steam spicules grew outward from the mounds with their apices pointing away from and perpendicular to their accretionary surfaces. In contrast, the wavy laminated biofabric of the dry mound sinter lacked well-developed spicules. We interpret the absence of the spicules on this part of the dry mound as an indication that, when the geyser was more active, fluid flow was likely more persistent and flowed over the sinter for longer periods of time during its formation.

### Microbial Transition Through Different Stages of Hydrothermal Activity at *El Tatio*

**FIGURE 8:**
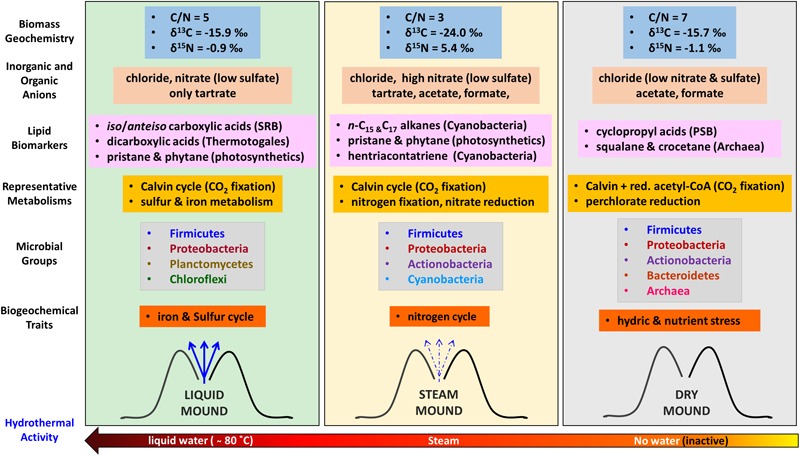
Schematic of the biogeochemical reconstruction of the three geothermal systems (liquid, steam, and dry) at *El Tatio* as a function of the hydrothermal activity based on bulk elemental and isotopic geochemistry, characteristic lipid biomarkers (source diagnosis in brackets), compound-specific isotopic analysis (metabolism), DNA sequencing (phylogeny), and LDChip immunoassays (phylogeny, metabolism, and biogeochemical traits). SRB means Sulfur Reducing Bacteria, GnSB Green non-Sulfur Bacteria, and PSB Purple Sulfur Bacteria. Red, acetyl-CoA stands for the reductive acetyl-CoA pathway for autotrophic CO_2_ fixation.

The different geochemical, mineralogical, and morphological features of the three sinter mounds were accompanied by distinct molecular (lipids and immuno-detections) and genomic biopatterns, which allowed us to describe a biogeochemical transition (prokaryotic-signatures based) along the hydrothermal activity gradient (Figure 8). Despite the ubiquitous dominance of Firmicutes and Proteobacteria in the three systems, the relative abundance of certain phylogenetic groups and specific biomarkers made a difference in the microbial composition at the three stages of hydrothermal activity. For instance, in the hydrothermally active liquid mound, the microbial community was observed to show a strong correspondence with phylogenetic groups such as Chloroflexi or Deltaproteobacteria. The detection of chlorophyll-derived lipids, such as pristane and phytane (Table 1), supported the contribution of photosynthetic microorganisms such as Chloroflexi to the community. Pristane and phytane are isoprenoid compounds mainly originating from phytol (Brocks and Summons, 2003), the esterifying alcohol of phototrophic chlorophylls (Didyk et al., 1978), although additional sources such as biphytane, archaeols or even tocopherol in the case of phytane, have been also described (Brocks and Summons, 2003). As for Proteobacteria, the detection by the LDChip200 of certain members such as Desulfuromonadales and *Desulfovibrio* sp., coincided with other against the Firmicutes *Desulfosporosinus* sp., suggesting the occurrence of sulfate reduction in the liquid, as well as steam, mound. In the liquid sample in particular, that detection agreed with the greater signal from sulfate reduction protein DsrA (Figure 7) and a larger detection of *iso/anteiso*-carboxylic groups (Figure 3) also associated with sulfate reducing bacteria (SRB), particularly the C_15_ and C_17_ pairs (Langworthy et al., 1983). In the absence of measurement of other sulfur species in the samples, the removal of sulfate by sulfate reducers (and/or less sulfate in the hydrothermal fluid at different times) would explain the lower concentration of this anion in the liquid sample (Figure 2A), accompanied by metabolic processes that utilized the only organic anion available to donate electrons in this geothermal system (*i.e.*, tartrate; Figure 2B). Low molecular-weight organic acids such as tartrate are excellent energy sources for anaerobic microbial metabolisms (e.g., Menes and Muxí, 2002; Maune and Tanner, 2012) such as sulfate reduction and methanogenesis (Parro et al., 2018).

Another distinctive molecular feature of the liquid mound was the larger concentration of dicarboxylic acids relative to the other two systems (Table 1), which may be attributed to the presence of thermophiles or hyperthermophiles, typically containing additional external protective membranes. Comparative distributions of dicarboxylic acids of slightly longer (C_16_ to C_22_) or much longer (C_30_ to C_32_) chains were described by Carballeira et al. (1997) in cultures of hyperthermophiles such as *Pyrococcus furiosus* and *Thermotoga maritima*, respectively. In the present study, the somewhat decreasing trend in concentration of the dicarboxylic acids from the liquid to the dry mound sample (Figure 3) was consistent with the selective disappearance of thermophiles from the hottest (*i.e.*, intermittent water at ∼80°C) to the coolest (*i.e.*, dry) setting.

Microbial metabolism in the liquid mound sample was dominated by the Calvin cycle, along with lesser sulfur and iron chemolithotrophic pathways. Autotrophic metabolism (Hayes, 2001) was supported by a large fractionation (*i.e.*, 11 to 26‰; Quandt et al., 1977; Preuβ et al., 1989) of δ^13^C values relative to atmospheric CO_2_ (ca. -8‰; Graven et al., 2017). Indeed, relatively light δ^13^C values (-21 to -36‰) were measured in the liquid sample for the carboxylic acids, *n*-alkanes, and *n*-alkanols (Figure 4). Complementarily, chemolithoautotrophs using sulfur-iron metabolism (Figure 7) were detected in different microenvironments, where heterotrophic metal (mainly iron) reducers would occupy anaerobic micro-regions, while sulfur and iron oxidizers would proliferate either in anaerobic or oxygen-rich microniches. In this case, the accessory minerals and dissolved ions would provide the metal and sulfur needed to support this community. Biofilms and EPS contributing to layering in the spicules observed in the sample (Supplementary Figure S1) may have aided to the isolation of these microenvironments.

In the steam mound, the strongest correspondence of the microbial community was observed with Cyanobacteria (Figure 5B). The relative enrichment of these microorganisms in this mound was consistently suggested by the relatively larger concentration of different cyanobacteria lipidic markers, such as mid-chain-length mono- and di-methyl alkanes (Dembitsky et al., 2001), octadecene (Shiea et al., 1991; Campbell et al., 2015), or unsaturated carboxylic acids (*i.e*., 16:1 ω7, 18:1 ω9, or 18:2 ω6; Cohen et al., 1995; Allen et al., 2010; Pagès et al., 2015; Table 1 and Supplementary Figures S6, S8). In agreement, the LDChip detected in this sample a relatively higher signal of biomaterial immunologically associated with *Anabaena* sp., *Leptolyngbya* sp., *Tolypothrix* sp., and *Chroococcidiopsis* sp. (Figure 7 and Supplementary Figure S9). In the steam system, Cyanobacteria seem to play an important role in the metabolism that supports the microbial community and possibly in the structures that formed under intermittent drier conditions.

Metabolically speaking, the steam sinter mound appeared largely influenced by the nitrogen cycle. The relatively larger immuno-signals of nitrogen fixation proteins (Nif and related Chaperons such as the HscA protein; Figure 7), together with the highest concentration of nitrate in the steam mound extract (Figure 2A), indicated that organisms capable of N_2_ fixation and subsequent initiation of nitrification, such as *Cyanobacteria* or the alphaproteobacterial order *Rhizobiales*, were active in the hydrothermally intermittent system. Additionally, the presence of the nitrate reductase protein (NRA in Figure 7) suggested that nitrate-reducing microorganisms could be making the most of the nitrate as an energetic source to thrive in the steam mound. Oxidation of organic matter (Chin and Janssen, 2002) or other inorganic compounds (*e.g.*, H_2_ or sulfides) would assist with nitrate reduction. The distinctly high nitrate concentration in the steam sinter extract corresponded to morphological changes in the biofilms that developed in the interstices between the spicules. This shift in the microbial population is supported by the change to highly porous and silicified remnants observed in the interstices (Supplementary Figure S2). The presence of nitrate would also serve as a substrate for denitrification processes, which would be consistent with the highest detection of nitrate reductase (Figure 7) and the exclusive detection of nitrite in the steam sinter (Supplementary Table S2). In either case, the molecular evidence indicated that, together with autotrophic Calvin pathways (Figure 4), nitrogen cycle was a crucial metabolic trait in the microbial biofilm of the steam mound, where nitrate appeared as a central metabolite of different, co-occurring metabolic pathways.

The microbial community in the dry mound showed a strong correspondence with Archaea, Gammaproteobacteria, and Actinobacteria (Figure 5B). The relative abundance of Actinobacteria, a phylum with great adaptability to aridity and resistance to hydric stress and UV radiation (Makarova et al., 2001), coincided with the positive immuno-detections of proteins related to hydric stress (*i.e.*, DhnA1 peptide from a dehydrin protein; Supplementary Figure S9), consistent with the limited access to water in the inactive geothermal system. In addition, the LDChip detected compounds indicative of nutritional stress conditions such as PHAs (Figure 7), which are produced upon need for carbon storage under nutritional stress or high C/N ratios (Steinbüchel, 1991; Byrom, 1994). The episodic waning of geyser activity in the history of sinter accretion coincides with the scarcity of water and bioavailable nutrients, which would subsequently lead to hydric and nutritional stress. Indeed, the most abundant Firmicutes orders in the dry mound (*i.e.*, Bacillales and Clostridiales) corresponded to microorganisms characteristically resistant to extreme conditions (e.g., desiccation and oxidative stress), including those able to form endospores (Paredes-Sabja et al., 2011) as an adaption for desiccation and oxidative stress.

Gammaproteobacteria was also particularly representative of the dry-mound microbial community (Figure 5B). Within them, purple sulfur bacteria (PSB) appeared to be present, according to the detection of low concentration (Table 1) of C_17_ and C_19_ cyclopropyl acids (Bühring et al., 2014) with δ^13^C values (Figure 4D) in the range of those assigned to PSB (from -20 to -29‰) in microbial mats from, e.g., Shark Bay (Pagès et al., 2015). Cyclopropane carboxylic acids are bacterial membrane components typically transformed from unsaturated carboxylic acids when exposed to stressful conditions such as oxidants, starvation, or desiccation (Grogan and Cronan, 1997; Chen and Gänzle, 2016). Their formation decreases the permeability of bacterial membranes, enhancing their stability under environmental stress (Poger and Mark, 2015). Wilhelm et al. (2018) reported detection of these carboxylic acids in surface soils from hyperarid regions of the Atacama Desert at about 430–480 km southwest of the *El Tatio* geysers field (Chañaral and Altamira). In the present study, their detection only in the dry sample may be a response to an adaptive strategy against desiccation and exposure to higher radiation in the inactive sinter geyser.

The closest correspondence of the dry sample was with Euryarchaeota and Crenarchaeota (Figure 5B). The contribution from archaeal sources solely in the dry mound was consistent with the detection of squalane and crocetane only in that sample (Table 1), respectively, attributed to halophylic (ten Haven et al., 1988) or methanogenic/methanotrophic archaea (Brocks and Summons, 2003). The lack of archaeal signal in the dry sample by the LDChip200 was attributed to intrinsic limitations on the technique, in relation to the absence of antibodies against the specific archaeal strains detected here by DNA sequencing. The LDChip200 is an assay interrogating a panel of 181 polyclonal antibodies (*i.e.*, close method), in contrast to the ability of the DNA sequencing in detecting any existing phylogenetic group (*i.e.*, open method). Furthermore, the antibodies used in the LDChip were produced using whole cell lysates of particular strains or whole EPS fractions as immunogens. This means that each polyclonal antibody preparation may contain subpopulations of antibody molecules recognizing their target with different specificities and affinities. They can bind epitopes from a variety of microorganisms, not necessarily the same species, but related ones, or even to others well conserved among large phylogenetic groups. Consequently, a direct correlation at species level between LDChip and DNA sequences should not be expected. This is a drawback of the LDChip, yet it increases the chances for detecting any microbial remains in life detection experiments.

Finally, certain occurrence of perchlorate reduction activity was considered in the dry mound, as suggested by the LDChip200 detection against Proteobacteria capable of perchlorate reduction such as *Magnetospirillum* sp., *Ideonella* sp, and *Dechlorobacter* sp. (Supplementary Figure S9). Despite the detection of markers by the LDChip, the presence of perchlorate reducing indicators in the dry sinter should be further investigated, since no perchlorate anions were detected in the samples. Whether these microorganisms are indeed using minor amounts of perchlorate as electron acceptor (Nepomnyashchaya et al., 2012) or they are only detoxifying photochemically generated chloride species (Trumpolt et al., 2005) has to be determined. As for CO_2_ fixation, the consistent depletion (∼2–4‰) of the δ^13^C ratios in the majority of lipid compounds in the dry sample relative to the other two mounds (Figure 4) suggested a shift in the metabolic fingerprint that could be related to water scarcity. Even though the Calvin cycle may still dominate the autotrophic metabolism in the dry sample (δ^13^C values from -24 to -32‰), the general decrease in compound-specific δ^13^C ratios may be caused by some contribution from assimilation pathways with larger ^13^C fractionations (*i.e.*, reductive acetyl-CoA pathway; Fuchs, 1989; Preuβ et al., 1989).

The microbial community in the three sinter mounds showed a transition through the different stages of hydrothermal activity, with the dry mound displaying generally larger alpha-diversity estimates (Figure 6), that is, a richer, more diverse and even population. Although the opposite trend was observed in some of the LDChip-based estimates (i.e., richness and Shannon’s diversity), the discrepancy was attributed to known factors, such as (i) the differences in approach underlying the LDChip200 and DNA sequencing techniques (*i.e.*, closed versus open methods), and (ii) the fact that the drier the sample, the lower the biomass to analyze, which can reduce the concentrations of many target molecules to values below the limit of detection for the LDChip200. The consistently greatest Pielou’s index values in the dry sample illustrated the impact of the decreasing hydrothermal activity in the microbial community structure. Greater evenness is generally correlated with less active communities and is consistent with the dormant stage of the dry sinter mound (*i.e.*, inactive geyser). A hydrothermally active geyser, such as the liquid water-engulfed sinter mound at *El Tatio*, is initially colonized (primary community) by microorganisms able to endure and thrive at high temperatures (*i.e.*, thermophiles and hyperthermophiles). The reduced group of taxa capable to establish in such high temperature results in a microbial community of low richness, diversity and evenness. As hydrothermal activity wanes and dry periods lengthen (i.e., the steam-wetted and dry sinter mounds at *El Tatio*), temperature ceases to be a key factor and thermophiles became less competitive compared to other microbial communities. Opportunistic microorganisms comprising endoliths and communities contributed by environmental contaminants emerge overprinting the primary thermophiles (secondary community). While approaching dormancy, the inactive, dry geyser displayed the richest, most diverse and most even microbial community composed of members and metabolisms that survived the waning of geyser activity.

## Conclusion

The multidisciplinary field and molecular study was successful in explaining the influence of the degree of hydrothermal activity on the biomarkers record (i.e., including present and past biosignatures) in the three morphologically similar geyser mounds at *El Tatio*. In the context of the ecological and environmental setting, the phylogenetic, molecular, and metabolic patterns, in agreement with differences in the micron-scale morphology of the geyserite, followed a hydrodynamic gradient predictable during the lifetime of a geyser mound; the transition from persistent hydrothermal activity to intermittent steam exposure to episodic and eventually, continual dryness. Accordingly, the microbial population shifted from primary communities adapted to high temperature (thermophiles and hyperthermophiles) to populations adopting protective strategies against desiccation and increased exposure to intense UV radiation as geyser activity waned. Alpha-diversity estimates supported the transition toward a richer and more even microbial population with the waning of the hydrothermal activity. Our results demonstrated the effectiveness of integrating microbiological and biogeochemical approaches to document and understand the microbial community structure and function in high-altitude geothermal environments with resemblance to Hesperian surfaces on Mars. Gathering data about the capability of different analytical techniques to decipher information from preserved fossil biosignatures is of vital importance for future astrobiological missions.

## Author Contributions

DC, NC, KW-R, and NH collected the sinter samples. DC and LS-G extracted and analyzed the lipidic fractions and wrote the manuscript. MF-M, SP, KL, KW-R, MB, and DL-B extracted DNA performed DNA sequencing and discussed phylogenetic results. MG-V, YB, and VP performed and analyzed the LDChip200 immunoassays. MG-V and VP performed ion chromatography analysis. VP, SC, NH, KW-R, and SP contributed to improve the manuscript edition.

## Conflict of Interest Statement

The authors declare that the research was conducted in the absence of any commercial or financial relationships that could be construed as a potential conflict of interest.

## Footnotes

1 http://www.ncbi.nlm.nih.gov/sra
