# Supplementary material for: Microbial Biomarker Transition in High-Altitude Sinter Mounds From El Tatio (Chile) Through Different Stages of Hydrothermal Activity
Source: Front Microbiol. 2019 Jan 15;9:3350. doi: 10.3389/fmicb.2018.03350 (PMC6340942; doi:10.3389/fmicb.2018.03350)
Supplement: Supplementary file 1 [file Data_Sheet_1.docx]

**Supplementary Information**

This section contains 1 supplementary text, 3 tables and 9 figures**.**

**Supplementary Text S1. Analysis of microarray data and selection of a cutoff .**

The selection process of data produced from the LDChip immunoassay involved the following key standard steps:

1. Those spots having obvious defects (tiny spots, missing signal, or artifacts due to a dust on the microarray or a bad wash) were removed and not considered for quantification.
2. The final fluorescence intensity (F) for each antibody spot is calculated with the following equation:

F=(F635-B)sample – (F635-B)blank

Where F635 is the median of the fluorescence of all the spot pixels at 635 nm and B the local background around the spot (which is quantified by the software), either for the sample or the blank control. A blank is always run in parallel with only buffer as an “antigenic” sample and then revealed with the same fluorescent antibody cocktail as the real samples. Those positive spots obtained after incubation of the array with only the fluorescent antibody cocktail are considered to be false positives and are removed from the analysis.

1. To minimize the probability of false positives, we increase the level of stringency by applying to all spots an additional cutoff value of 2.5-fold the average of F of the whole array.

The cutoff is usually an arbitrary value that depends on the quality and distribution of the data set. In the literature there are different examples of the multiple methods existing to define a cutoff, e.g.: (1) those based on the signal-to-noise ratio (SNR) (SNR=signal intensity-background/standard deviation of background), where a SNR cutoff value of 2 is commonly accepted for environmental samples (He et al., 2007); (2) those using the averaged fluorescent signal provided by control spots or spots that should not exhibit any fluorescent signal (e.g. spots only containing BSA, buffer, or preimmune antiserum in antibody microarrays) (Parro et al., 2011), where cutoff values of 2-3 times the fluorescence intensity are normally used to discard false positives; (3) those determining cutoff values by using cumulative frequency histograms, where spots with F higher than a certain accumulated frequency (normally between 80% and 90%) and a subsequent increase lower than 10% are considered positives; or (4) those estimating cutoff values by as the average F in the whole array (Fernández-Remolar et al., 2013).

In the present study, we applied a cutoff value of 2.5-fold the average of F, based on the application of the 4^th^ method explained above and supported by its previous use in multiple studies (e.g. Blanco et al., 2015, 2017, 2019). Lacking of a systematic and unified criterion to define a cutoff, we wanted to be as restrictive as possible here in pursuit of the data quality. Thus, we increased the stringency by applying to all spots a cutoff value of 2.5-fold the average of F of the whole array, which we have observed to be the most restrictive when compared to those obtained with other methods.

**Table S1**. List of the antibodies added to the LDChip200 for this study. See Table S1 in Sánchez-García et al. (2018) for the complete set of antibodies printed on the LDChip200.

| **No** | **Ab name** | **Source/ Strain** | **Sample/ Culture conditions** |  | **Immunogen / Fraction** | **References** |
| --- | --- | --- | --- | --- | --- | --- |
|  |  |  |  |  |  |  |
| 213 | **IVK19C1** | *Chroococcidiopsis* sp. CCMEE 029 | BG11 |  | Whole cells | This work* |
| 214 | **IVL2C1** | *Magnetospirillum bellicus* VDY | Basal-phosphate buffer, acetate, chlorate |  | Whole cells | This work** |
| 215 | **IVL3C1** | *Ideonella dechloratans* | Basal-phosphate buffer, acetate, chlorate |  | Whole cells | This work** |
| 216  217 | **IVL4C1**  **p-IVL3C1** | *Dechlorobacter hydrogenophilus* LT-1  Pre-immune serum | Basal-phosphate buffer, acetate, chlorate  IgG fraction (protein A purified) |  | Whole cells  Pre-immune serum | This work**  This work |

**Chroococcidiopsis* cells were provided by Dra. Daniela Billi, University of Rome Tor Vergata, Italy.

***Magnetospirillum*, *Ideonella* and *Dechlorobacter* cells were provided by Dr. John D. Coates, Berkeley University, California (USA).

**Table S2**: Concentration of inorganic and organic anions (μg g^-1^) in the sinter samples from the three geyser mounds at *El Tatio* (liquid, steam, dry), and the water sample from the liquid mound.

|  | Liquid mound | |  | Steam mound |  | Dry mound |
| --- | --- | --- | --- | --- | --- | --- |
|  | water | sinter |  | sinter |  | sinter |
| Chloride | 7068 | 612 |  | 596 |  | 831 |
| Nitrate | n.d. | 457 |  | 1497 |  | 20 |
| Sulfate | 346 | 14 |  | 44 |  | 36 |
| Nitrite | n.d. | n.d. |  | 0.52 |  | n.d. |
| Fluoride | 12 | 6.2 |  | 4.3 |  | 19 |
| Bromine | 56 | n.d. |  | n.d. |  | n.d. |
| Tartrate | 286 | 1.1 |  | 7.2 |  | n.d. |
| Acetate | 0 | n.d. |  | 2.3 |  | 3.1 |
| Formate | 0 | n.d. |  | 2.2 |  | 2.4 |
| Propionate | n.d. | n.d. |  | n.d. |  | n.d. |
| Oxalate | n.d. | n.d. |  | n.d. |  | n.d. |

n.d. means not detected

**Table S3.**  Compound specific isotopic composition (δ^13^C) of alkanes, carboxylic acids, and alkanols in the sinter samples from the three mounds at *El Tatio* (liquid, steam, and dry).

|  | liquid | steam | dry |
| --- | --- | --- | --- |
| *alkanes* |  |  |  |
| *n*-C_16_ | -25.3 | -27.4 | -28.5 |
| *n*-C_17_ | -22.9 | -25.2 | -27.9 |
| *n*-C_18_ | -25.8 | -27.6 | -28.0 |
| *n*-C_19_ | -22.5 | -27.6 | -28.9 |
| *n*-C_20_ | -27.1 | -27.5 | -28.8 |
| *n*-C_21_ | -26.4 | -26.9 | -30.2 |
| *n*-C_22_ | -28.1 | -26.9 | -30.2 |
| *n*-C_23_ | -27.1 | -27.8 | -30.9 |
| *n*-C_24_ | -26.9 | -27.8 | -30.2 |
| *n*-C_25_ | -26.7 | -28.2 | -30.4 |
| *n*-C_26_ | -28.3 | -25.9 | -30.1 |
| *n*-C_27_ | n.a. | -25.2 | -30.3 |
| *n*-C_28_ | n.a. | -26.1 | -29.8 |
| *n*-C_29_ | n.a. | -26.7 | -31.3 |
| **Total *n*-alkanes** | **-26.1** | **-26.9** | **-29.7** |
| *carboxylic acids* |  |  |  |
| *n*-C_14_ | -23.9 | -24.1 | -25.3 |
| *n*-C_15_ | -25.6 | -26.9 | -27.4 |
| *n*-C_16_ | -25.3 | -25.6 | -25.8 |
| *n*-C_17_ | -20.5 | -22.8 | -24.1 |
| *n*-C_18_ | -24.9 | -24.5 | -25.1 |
| *n*-C_19_ | -22.5 | -24.8 | -25.3 |
| *n*-C_20_ | -27.4 | -27.3 | -27.1 |
| *n*-C_21_ | -23.8 | -24.5 | -25.1 |
| *n*-C_22_ | -30.9 | -32.1 | -30.9 |
| *n*-C_23_ | -29.6 | -29.0 | -28.8 |
| *n*-C_24_ | -32.9 | -34.1 | -32.0 |
| *n*-C_25_ | -31.6 | -33.0 | -29.2 |
| *n*-C_26_ | -36.1 | -34.9 | -32.3 |
| **Total *n*-carboxylic acids** | **-27.3** | **-28.0** | **-27.6** |
| isoC_15_ | -24.4 | -25.6 | -26.5 |
| anteisoC_15_ | -26.1 | -25.9 | -27.8 |
| isoC_17_ | -27.8 | -25.9 | -27.9 |
| anteisoC_17_ | -25.6 | -26.0 | -27.5 |
| isoC_18_ | -27.1 | -26.0 | -27.8 |
| anteisoC_18_ | -28.1 | -27.3 | -28.6 |
| **Total *iso/anteiso* carboxylic acids** | **-26.5** | **-26.1** | **-27.7** |
| Cyc-_17:0_  ^a^ | n.a. | n.a. | -24.4 |
| Cyc-_19:0_  ^b^ | n.a. | n.a. | -24.0 |
| C_18:1_ ^c^ | -27.5 | -27.9 | -28.0 |
| C_19:1_ ^d^ | -31.3 | -29.0 | -29.0 |
| *alkanols* |  |  |  |
| *n*-C_14_ | -27.4 | -27.5 | -27.6 |
| *n*-C_16_ | -27.7 | -27.8 | -28.4 |
| *n*-C_18_ | -24.8 | -27.7 | -30.2 |

^a^ Cyclopropyl heptadecanoic acid, with precise position of cyclopropyl unknown.

^b^ Cyclopropyl nonadecanoic acid, with precise position of cyclopropyl unknown.

^c^ Octadecenoic acid, with precise position of unsaturation unknown.

^d^ Nonadecenoic acid, with precise position of unsaturation unknown.

n.a. means not analyzed (due to low signal for the isotopic analysis in the GC-IRMS analysis)


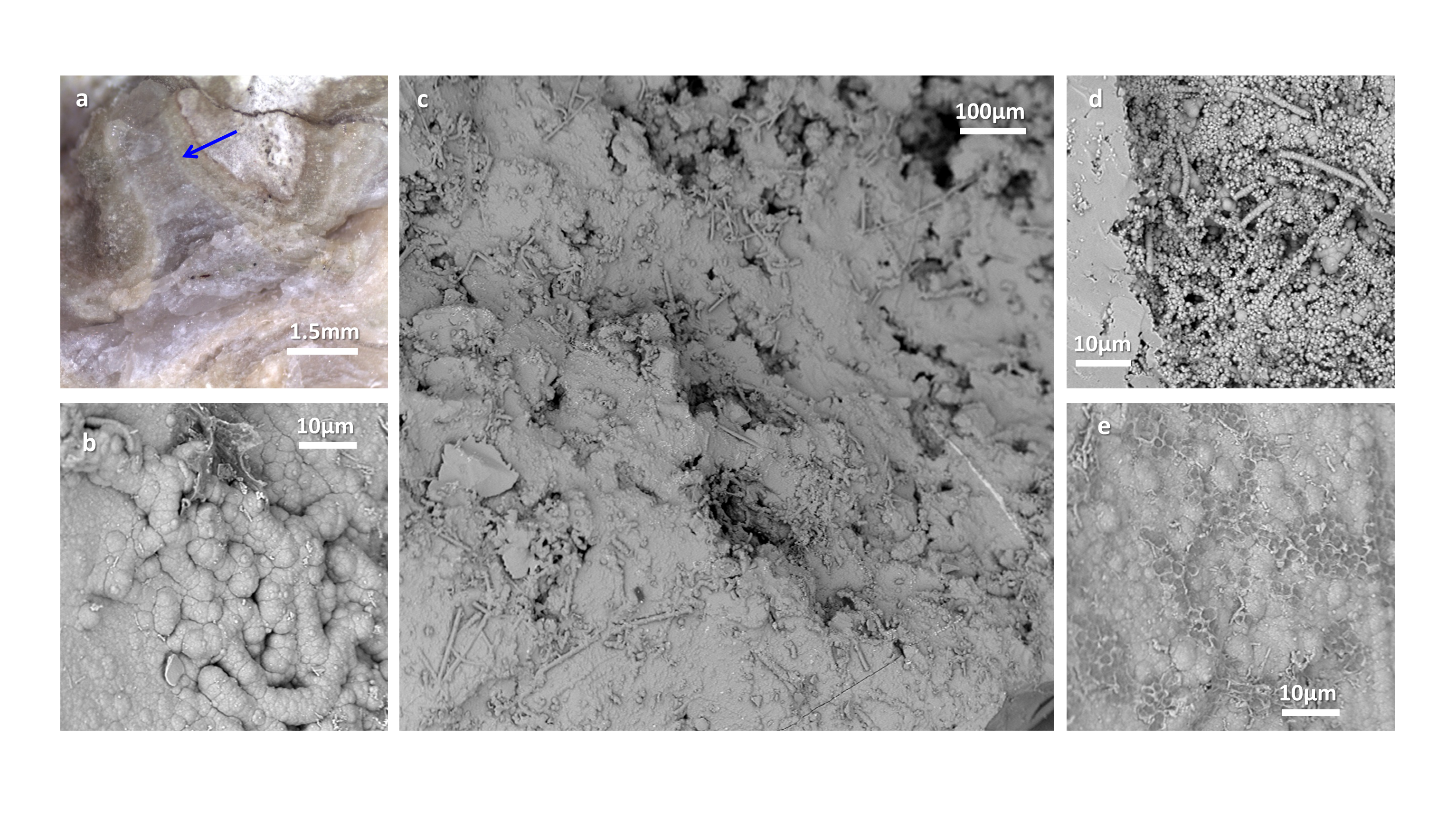


**Figure S1.** Stereoscope (a) and SEM (b-e) images of the sinter sample from the liquid mound at *El Tatio*; a) cross-sectional stereoscopic view of nearby spicules, where the blue arrow points at the outermost opaque and tan laminations of a spicule showing a downward parabolic orientation; SEM images of the top surface showing b) heavily silicified and c) less-silicified filament remnants; and SEM images of the sample inside showing d) cellular remnants entombed in opal-A nanocolloids, and e) extracellular remnants of honeycomb-like structure (i.e.exopolysaccharides, EPS).


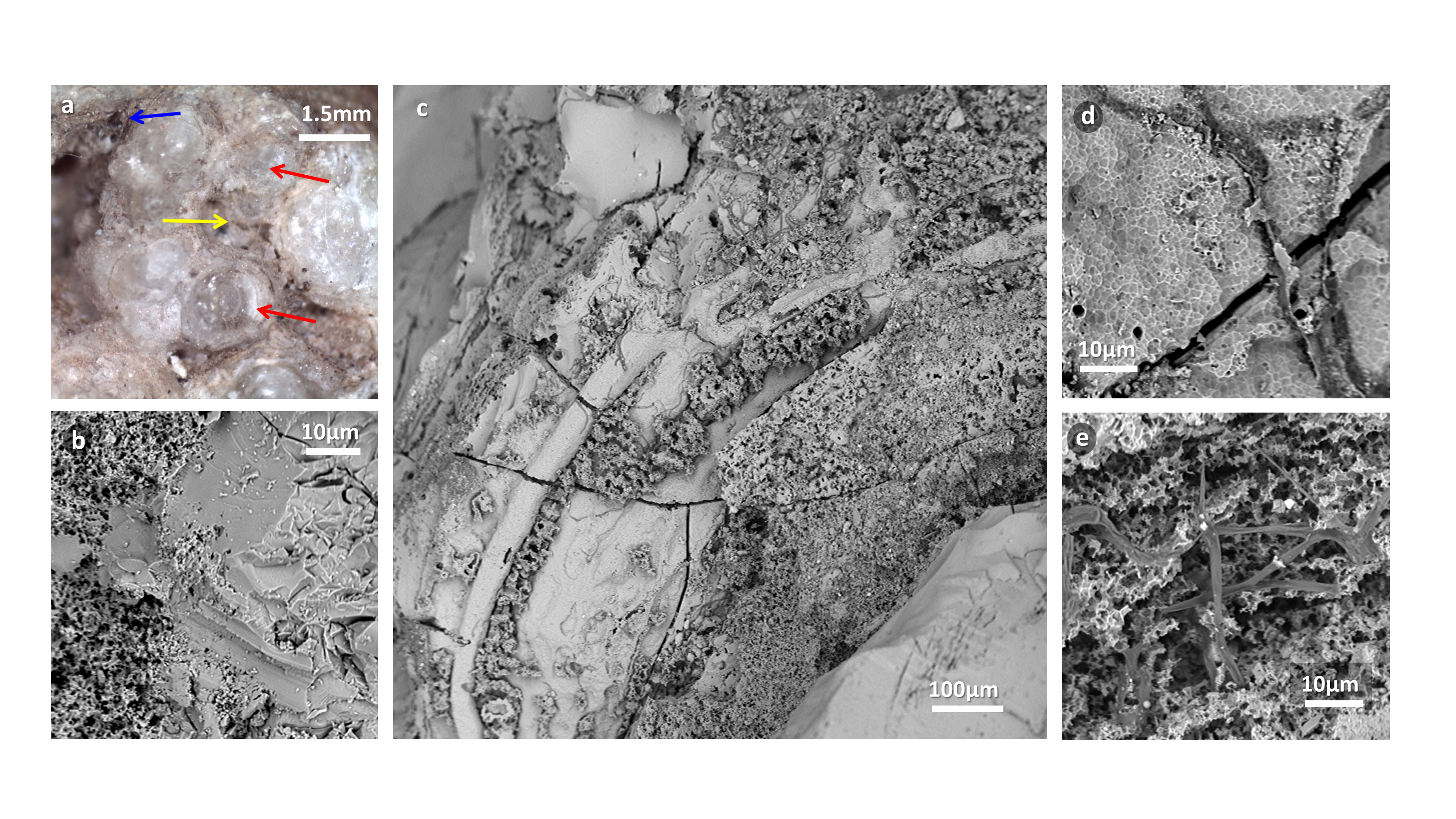


**Figure S2.** Stereoscope (a) and SEM images (b-e) of the sinter sample from the steam mound at *El Tatio*; a) top-down stereoscopic view of closely spaced spicules (red arrows) surrounded by silicified biofilms (yellow arrow) and detrital grains (blue arrow); SEM images of the top surface showing b) the interface between the highly porous silicified biofilm and massive vitreous opal-A; c) the outer edge of a spicule separated from the sample; d) cellular remnants of bacteria colonizing the spicular surfaces; and e) cellular remnants of bacteria within pore spaces of silicified remnants.


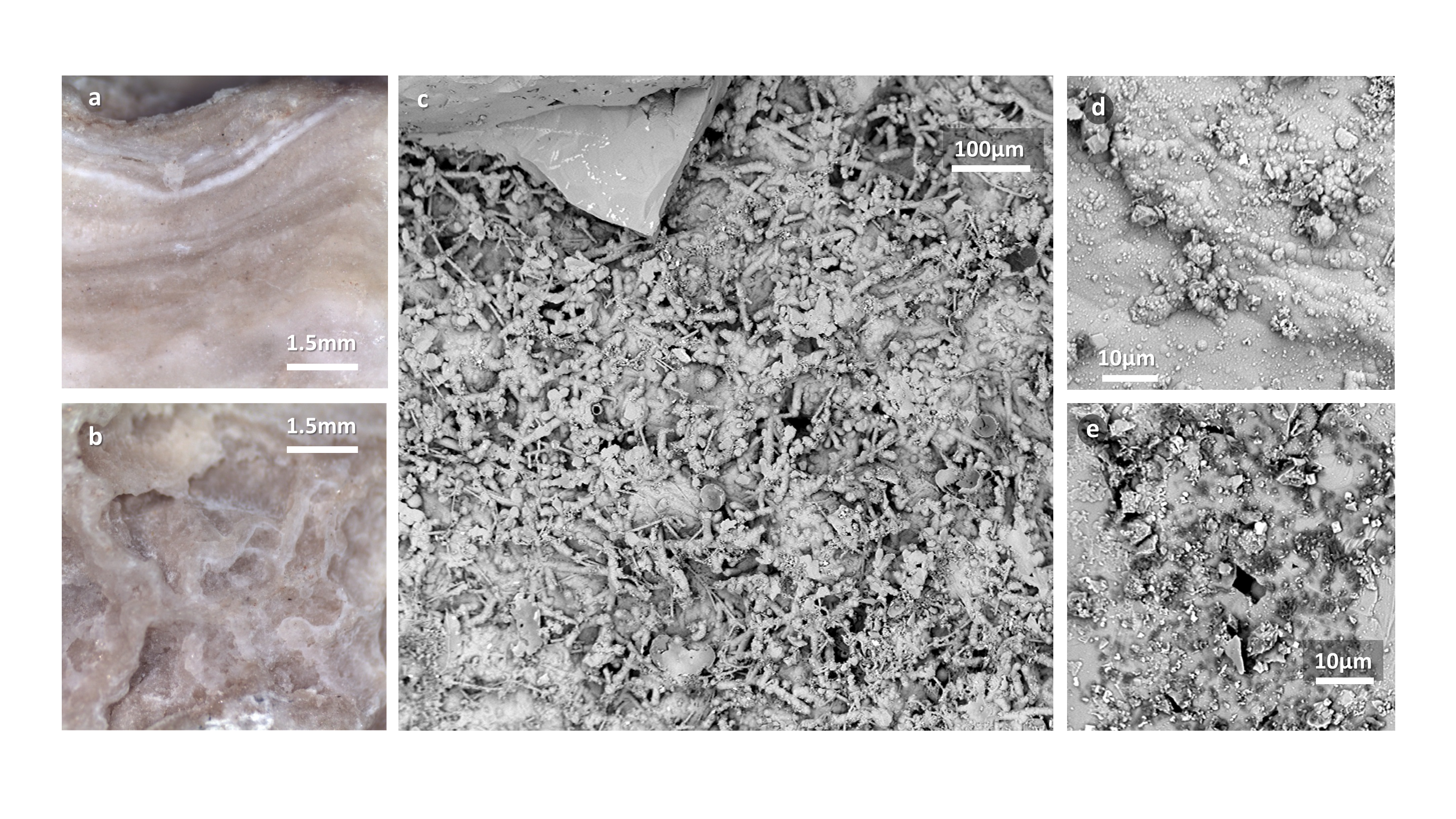


**Figure S3.** Stereoscope (a-b) and SEM images (c-e) of the sinter sample from the dry mound at *El Tatio*; a) cross-sectional stereoscopic view of wavy laminations of opal-A; b) stereoscopic plan view of an irregular network of ridges; SEM images of the sample surface showing c) well-preserved silicified remnants of a biofilm, including d) heavily entombed filaments, and d) organically preserved cocci remnants.


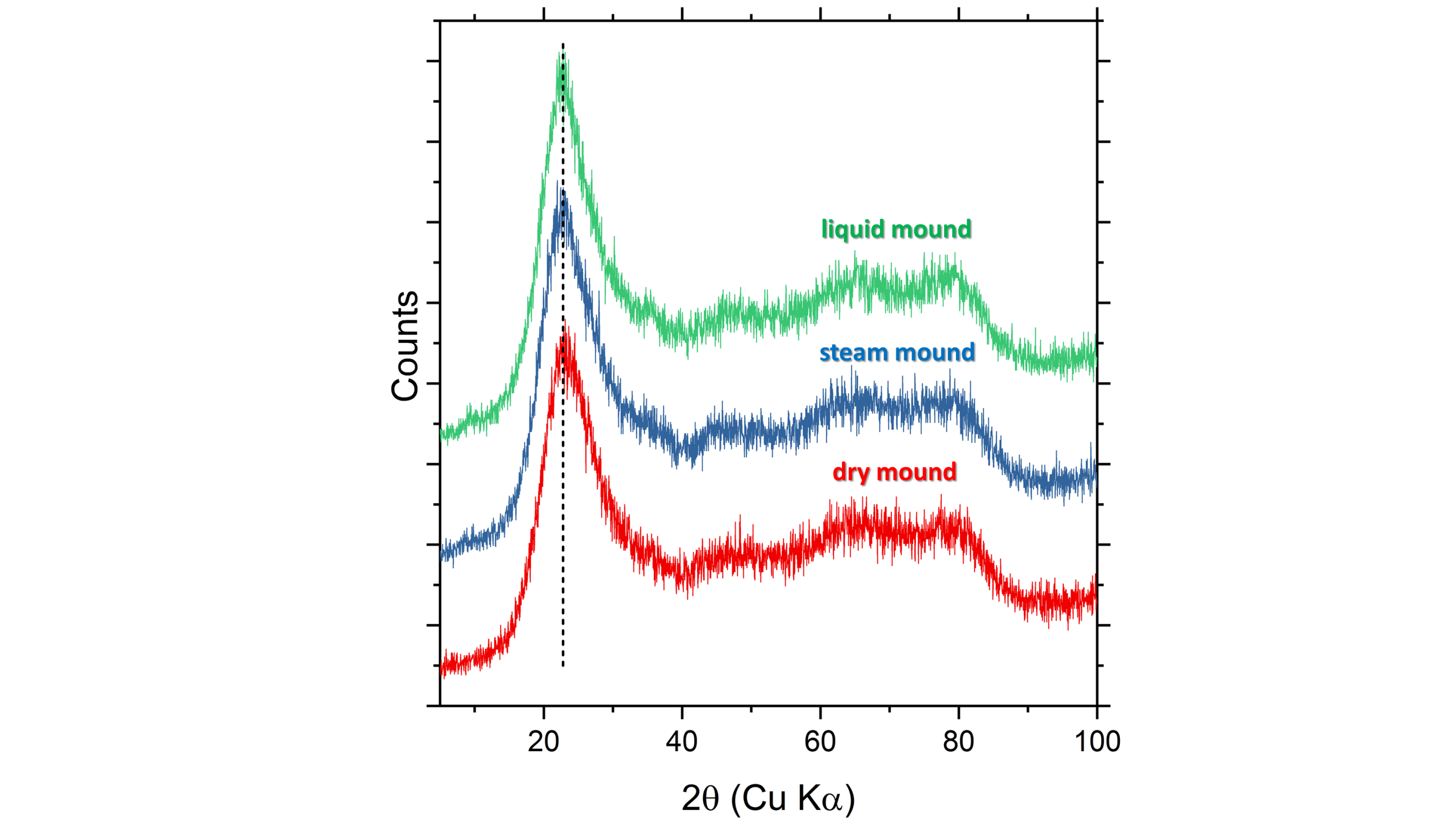


**Figure S4.** X-ray diffractograms produced by fragments of spicules and columns from the liquid (green) and steam (blue) mound samples, and from a non-porous, white region from the top of the dry sinter (red) (from analysis at PNNL). The dashed line points to the peak on the diffraction pattern of the three sinter samples (23° 2θ).

**
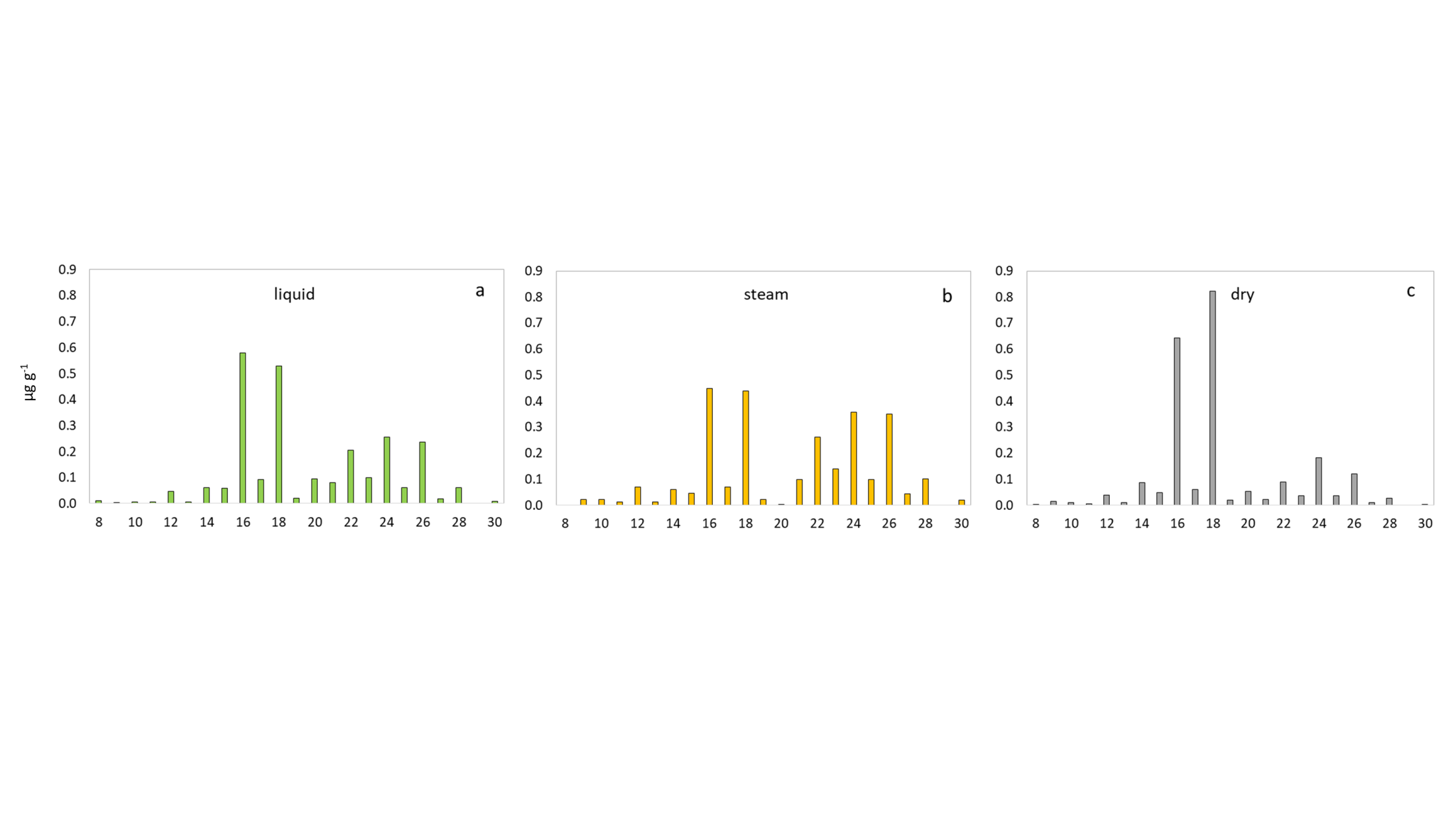
**

**Figure S5**. Molecular distribution patterns of *n-*carboxylic acids in the sinter samples from the three mounds at *El Tatio*; liquid (a), steam (b), and dry (c).


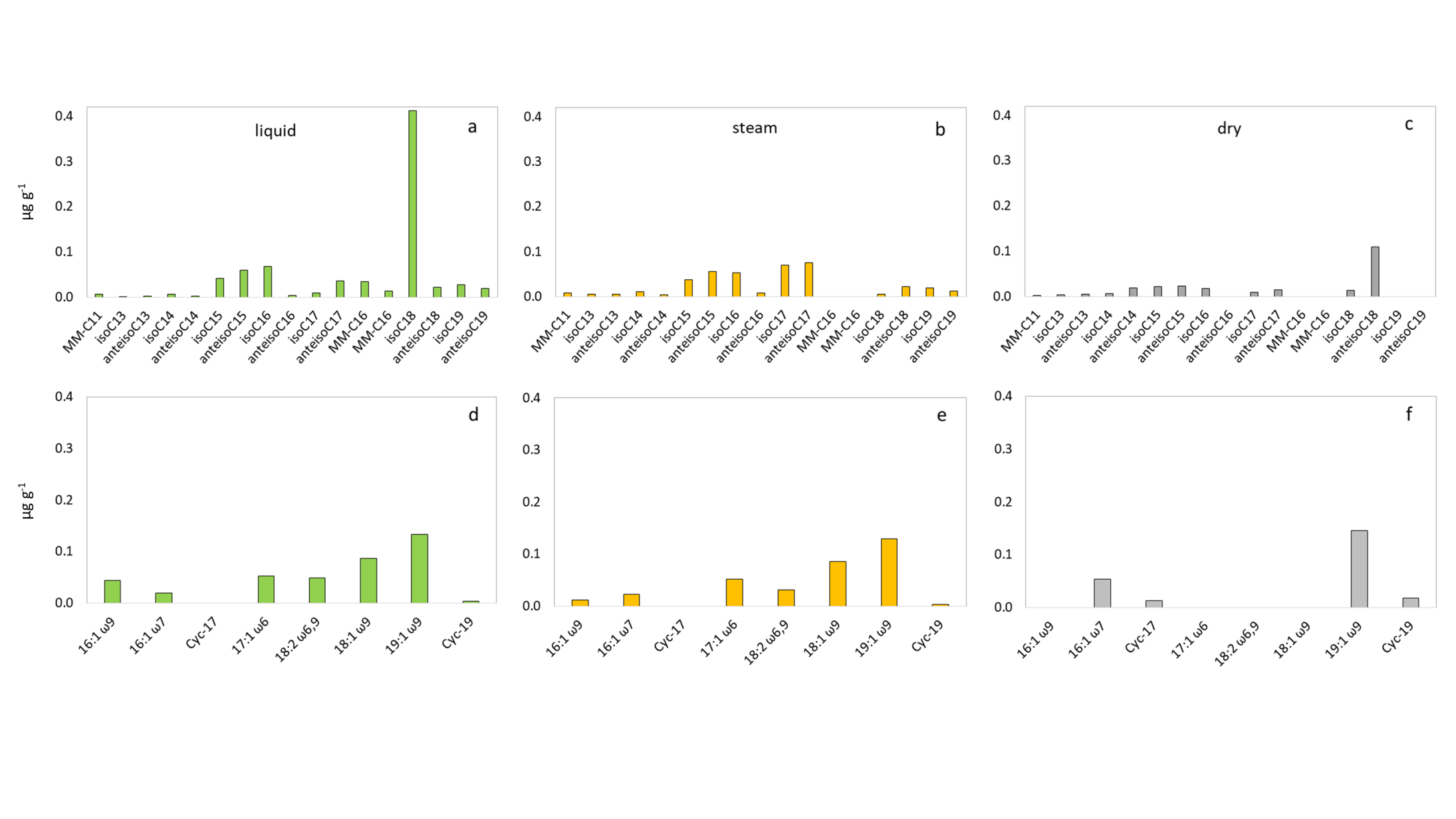


**Figure S6**. Molecular distribution of branched (a-c) and unsaturated (d-f) carboxylic acids in the sinter samples from the three mounds *at El Tatio*; liquid (green), steam (yellow), and dry (gray). In the branched acids panels (a-c), MM stand for mono-methyl carboxylic acids, whereas *iso*- and *anteiso*- represent mono-methyl branches at C-2 and C-3, respectively. In the unsaturated acids panels (d-f), the observed mono-unsaturation (n:1) are represented with the omega notation (i.e. counting the unsaturation position from the last carbon unit). Please note that two cyclopropane carboxylic acids (Cyc-17:0 and Cyc-19:0) are also displayed in the unsaturated acid panel.


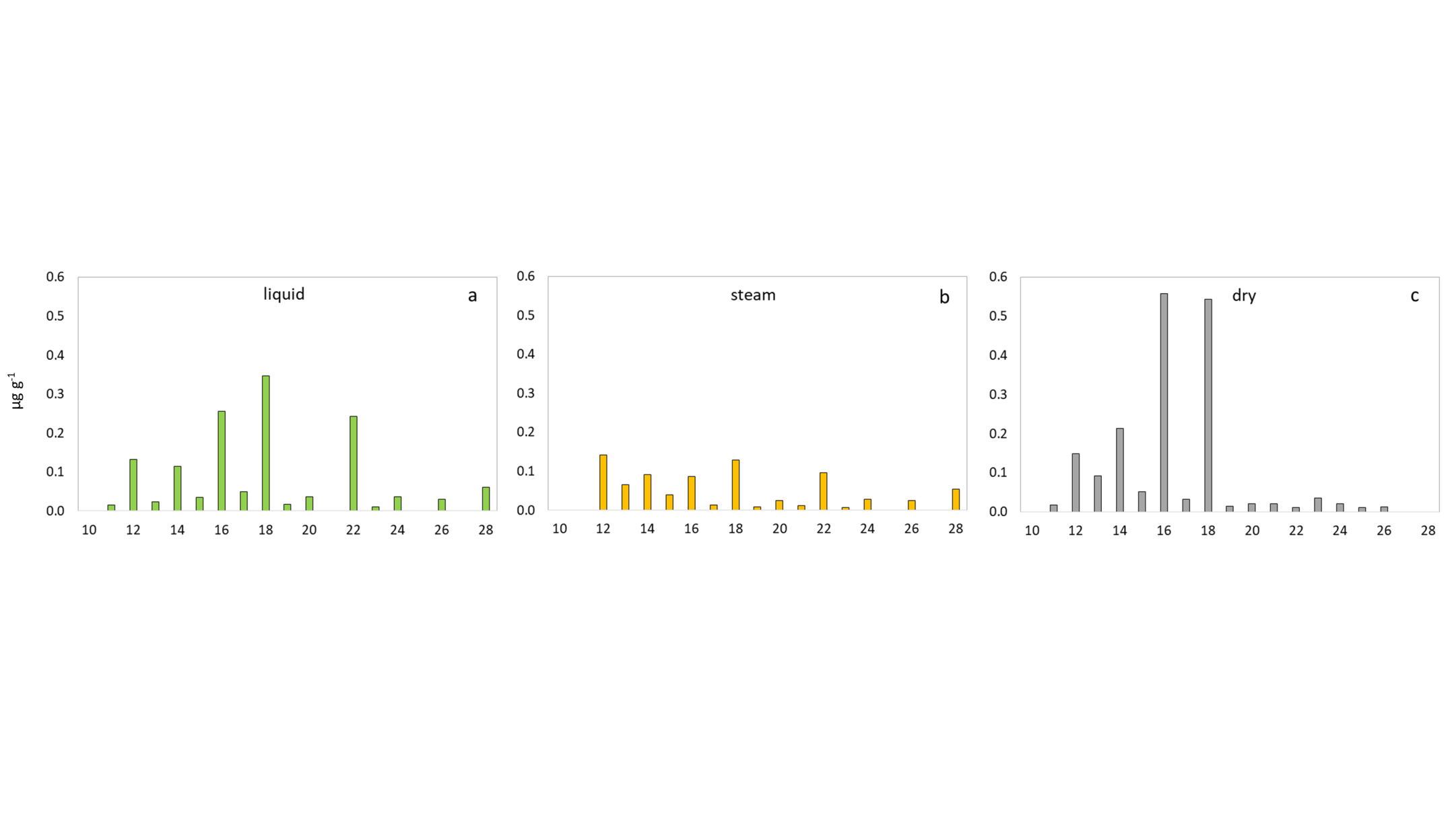


**Figure S7**. Molecular distribution patterns of *n*-alkanols in the sinter samples from the three mounds at *El Tatio*; liquid (a), steam (b), and dry (c).


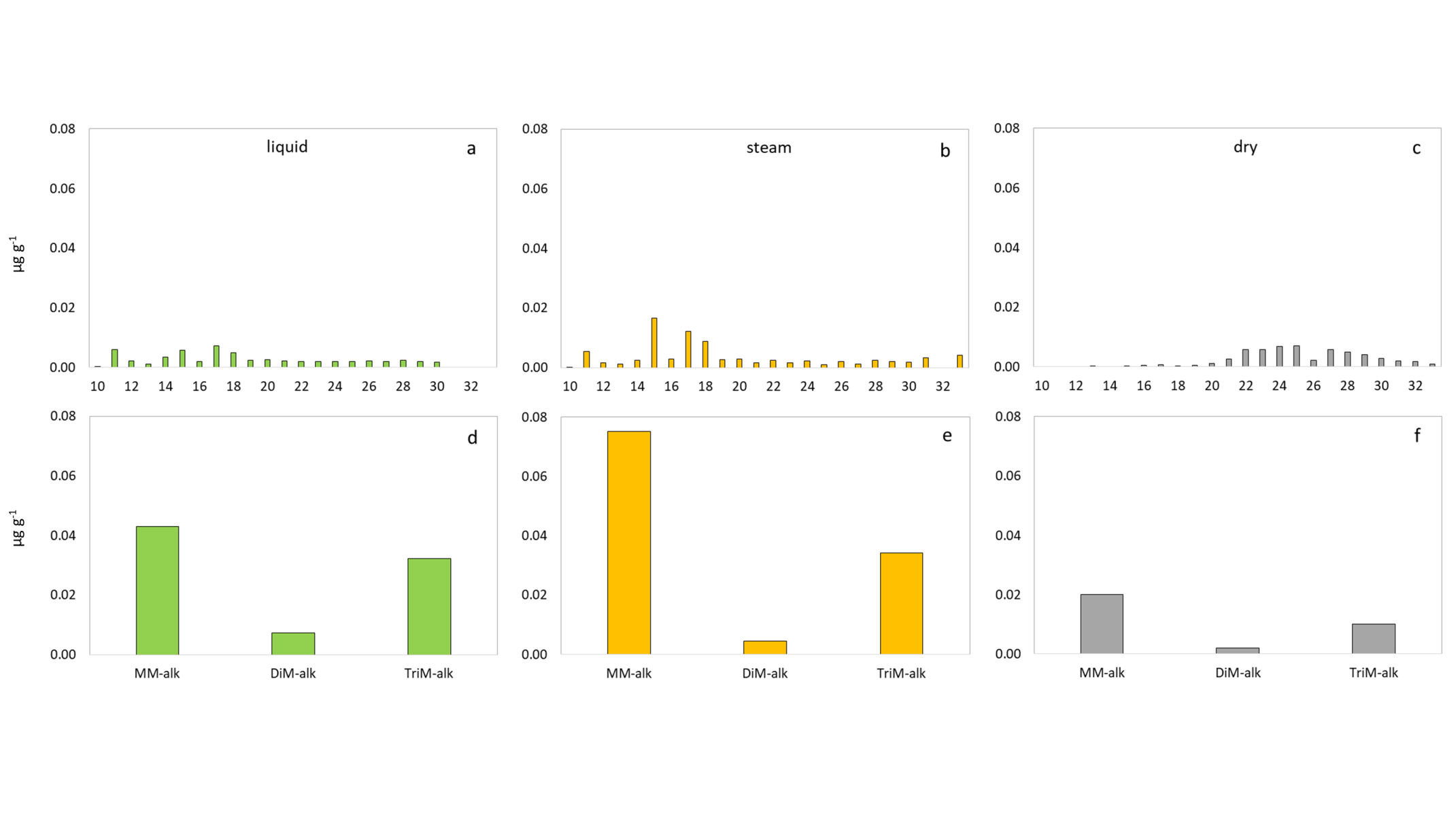


**Figure S8**. Molecular distribution of *n*-alkanes (a-c) and branched alkanes (d-f) in the sinter samples from the three mounds at *El Tatio*; liquid (green), steam (yellow), and dry (gray). The X-axis represents the number of carbon units in the a-c panels, whereas mono-methyl (MM), di-methyl (DM), and tri-methyl (TM alkanes in the d-f panels.

**
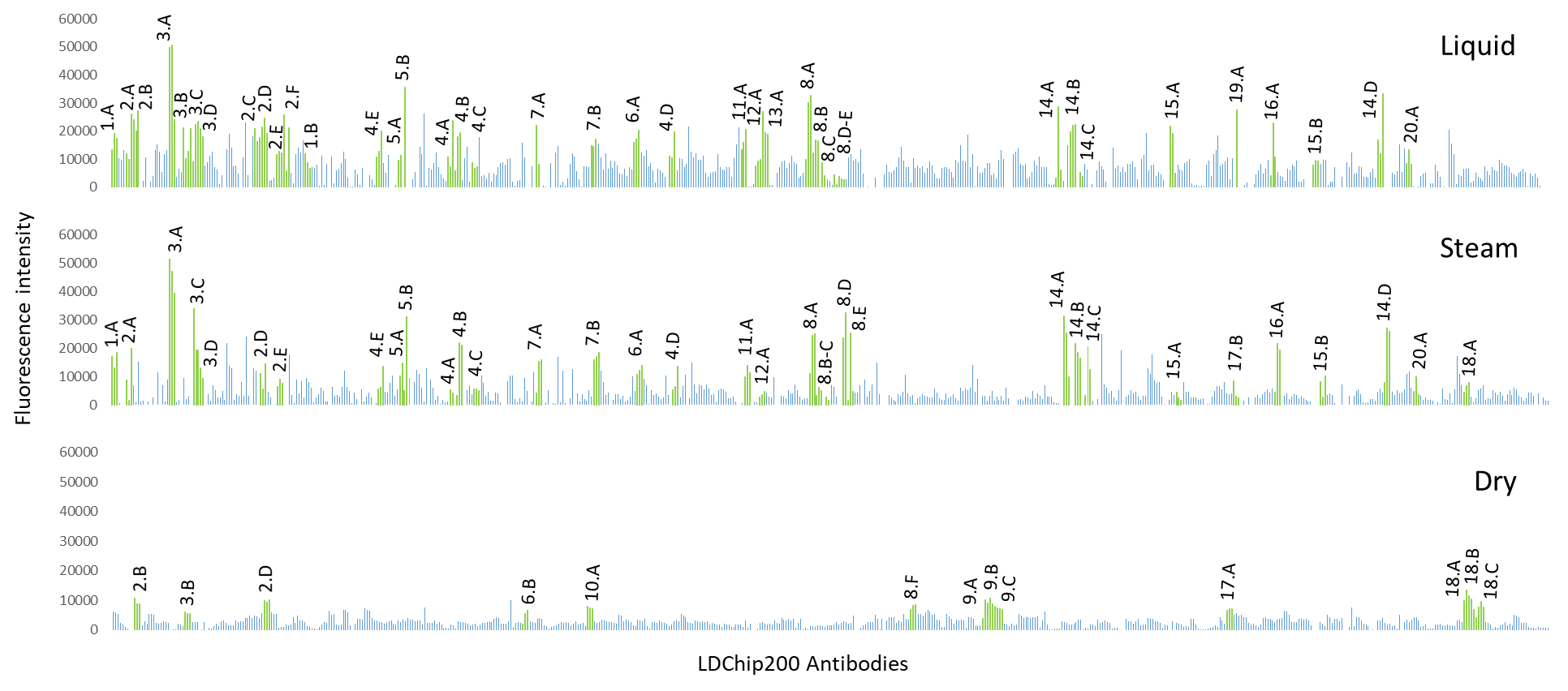
**

**Fig. S9**. Example of three immunograms after analyzing the sinter samples from the liquid, steam, and dry mounds with the LDChip200. Each antibody on the plot is represented by the 3 bars obtained by quantifying the fluorescence of the 3 spots per antibody in the LDChip200, after corrected by the blank control. In green, antibodies showing positives reactions against bacterial and archaeal biomarkers and conserved proteins, after corrected by the cutoff and the blank control (see the Material and methods section). The numbers indicate the categories in which the whole antibodies were clustered (see Fig. 7 for category information) and the letters the antibodies belonging to each category: 1.A: Antibody A-139, 1.B: IVE8C1, 2.A: A-183, 2.B: A-184, 2.C: IVE4C2, 2.D: IVE4S100, 2.E: IVE6C1, 2.F: IVE6C2, 3.A: IC1C1, 3.B: IC4C1, 3.C: IC6C1, 3.D: IC7C1, 4.A: IVG1C1, 4.B: IVG2C1, 4.C: IVG4C1, 4.D: IVI3C1, 4.E: IVF2C1, 5.A: IVF4C1, 5.B: IVF4S100, 6.A: IVI23C1, 6.B: IVH22C1, 7.A: IVI10C1, 7.B: IVI19C1, 8.A: IVK14C1, 8.B: IVK15C1, 8.C: IVK16C1, 8.D: IVK18C1, 8.E: IVK19C1, 8.F: IVK4C1, 9.A: IVL2C1, 9.B: IVL3C1, 9.C: IVL4C1, 10.A: IVI17C1, 11.A: IVJ4C1, 12.A: IVJ6C1, 13.A: IVJ8C1, 14.A: A-1483, 14.B: A-1485, 14.C: A-1487, 14.D: A-NifH1_12291, 15.A: A-bacteriorodopsin, 15.B: A-ICDH_11755, 16.A: A-GroEL AMS, 17.A: A-DhnA1, 17.B: A-DhnA2, , 18.A: A-PhaC1, 18.B: A-PhaC2, 19.C: A-PhcA1, 19.A: A-DsrA_11365, 20.A: A-NRA_11912. See Table S1 and Table S1 in Sánchez-García et al. (2018) for antibody information.
